# Auto3DCryoMap: an automated particle alignment approach for 3D cryo-EM density map reconstruction

**DOI:** 10.1186/s12859-020-03885-9

**Published:** 2020-12-28

**Authors:** Adil Al-Azzawi, Anes Ouadou, Ye Duan, Jianlin Cheng

**Affiliations:** Electrical Engineering and Computer Science Department, University of Missouri, Columbia, MO 65211 USA

**Keywords:** Cryo-EM, 3D density map, Particle alignment, Particle picking, Protein structure

## Abstract

**Background:**

Cryo-EM data generated by electron tomography (ET) contains images for individual protein particles in different orientations and tilted angles. Individual cryo-EM particles can be aligned to reconstruct a 3D density map of a protein structure. However, low contrast and high noise in particle images make it challenging to build 3D density maps at intermediate to high resolution (1–3 Å). To overcome this problem, we propose a fully automated cryo-EM 3D density map reconstruction approach based on deep learning particle picking.

**Results:**

A perfect 2D particle mask is fully automatically generated for every single particle. Then, it uses a computer vision image alignment algorithm (image registration) to fully automatically align the particle masks. It calculates the difference of the particle image orientation angles to align the original particle image. Finally, it reconstructs a localized 3D density map between every two single-particle images that have the largest number of corresponding features. The localized 3D density maps are then averaged to reconstruct a final 3D density map. The constructed 3D density map results illustrate the potential to determine the structures of the molecules using a few samples of good particles. Also, using the localized particle samples (with no background) to generate the localized 3D density maps can improve the process of the resolution evaluation in experimental maps of cryo-EM. Tested on two widely used datasets, Auto3DCryoMap is able to reconstruct good 3D density maps using only a few thousand protein particle images, which is much smaller than hundreds of thousands of particles required by the existing methods.

**Conclusions:**

We design a fully automated approach for cryo-EM 3D density maps reconstruction (Auto3DCryoMap). Instead of increasing the signal-to-noise ratio by using 2D class averaging, our approach uses 2D particle masks to produce locally aligned particle images. Auto3DCryoMap is able to accurately align structural particle shapes. Also, it is able to construct a decent 3D density map from only a few thousand aligned particle images while the existing tools require hundreds of thousands of particle images. Finally, by using the pre-processed particle images, Auto3DCryoMap reconstructs a better 3D density map than using the original particle images.

## Background

Cryo-EM (electron microscopy) has emerged as a major method for determining the structures of proteins, particularly large ones [1]. It freezes purified proteins in solutions and then uses the electron microscope to image the frozen film [2]. Typically, the cryo-EM method does not require a crystallization step. It can be applied to a wide range of proteins. During the cryo-EM, many 2D images of proteins are produced in varying orientations [3]. The 2D images are classified or clustered corresponding to the same orientation first. Then, to improve the signal-to-noise-ratio, the 2D images are aligned and averaged. Finally, the 3D volume (density map) is produced from the averaged 2D images [4]. A density map is a 3D grid in which each point has a certain density value. The density value reflects the electron density based on the corresponding point in the 3D space. Hundreds of thousands of the particle images (2D) are required to build and produce a 3D density maps of good quality [5, 6]. EMAN2 [7], RELION [8], and SPIDER [9] are the popular methods developed for 3D cryo-EM map reconstruction. An initial 3D model is required for these methods to build a decent 3D density map in addition to the manual particle picking issue.

In our approach, fully automated 3D density maps are constructed with no need for an initial 3D model. First, the set of particles is fully automatically picked, isolated, and selected as “good” and “bad” samples using our previous model DeepCryoPicker [10]. Second, the first stage of Auto3DCryoMap is designed to fully automate particle alignment. In our approach, instead of using the averaging process to summarize the similar particles in case of enhancing the contrast by increasing the signal-to-noise-ratio that helps in the alignment process and identifying bad or unwanted images (using a reference model), we use a new strategy. Our approach is based on using the unsupervised learning approach to generate a perfect binary mask (circular and square) for the top and side-view of particles. We design two fully automated approaches. The first one is designed fully automatically to align the binary mask of the square particle’s mask using the intensity-based image registration. Then, we project the angle’s difference between the original particle’s mask and the aligned one on the original particle. The second approach is designed for fully automated circular particle centralization. We used the same idea of the binary mask generation to produce a perfect binary circular mask for each particle. Then, our approach constructs the same center of each particle’s mask. A new particle dimension is reconstructed from the same center to build the same particle dimension. Finally, the second stage of the Auto3DCryoMap is designed for fully automated 3D density map reconstruction. Instead of using the common line or reference-based method for the 3D classification in addition to the pre-aligned steps that are required during the 3D construction step, we used a new approach that comprehends both. We designed a fully automated approach to build a localized 3D density map between every two aligned particles. First, the original particles are aligned using the intensity-based of the original particles not the binary mask for perfect alignment. Then, the 3D locations of the local matched points (corresponding) are estimated and calculated. Finally, the localized 3D density maps are projected together to produce the final 3D density map for each protein molecule.

## Methods

### Overview of the Auto3DCryoMap workflow

**Fig. 1:**
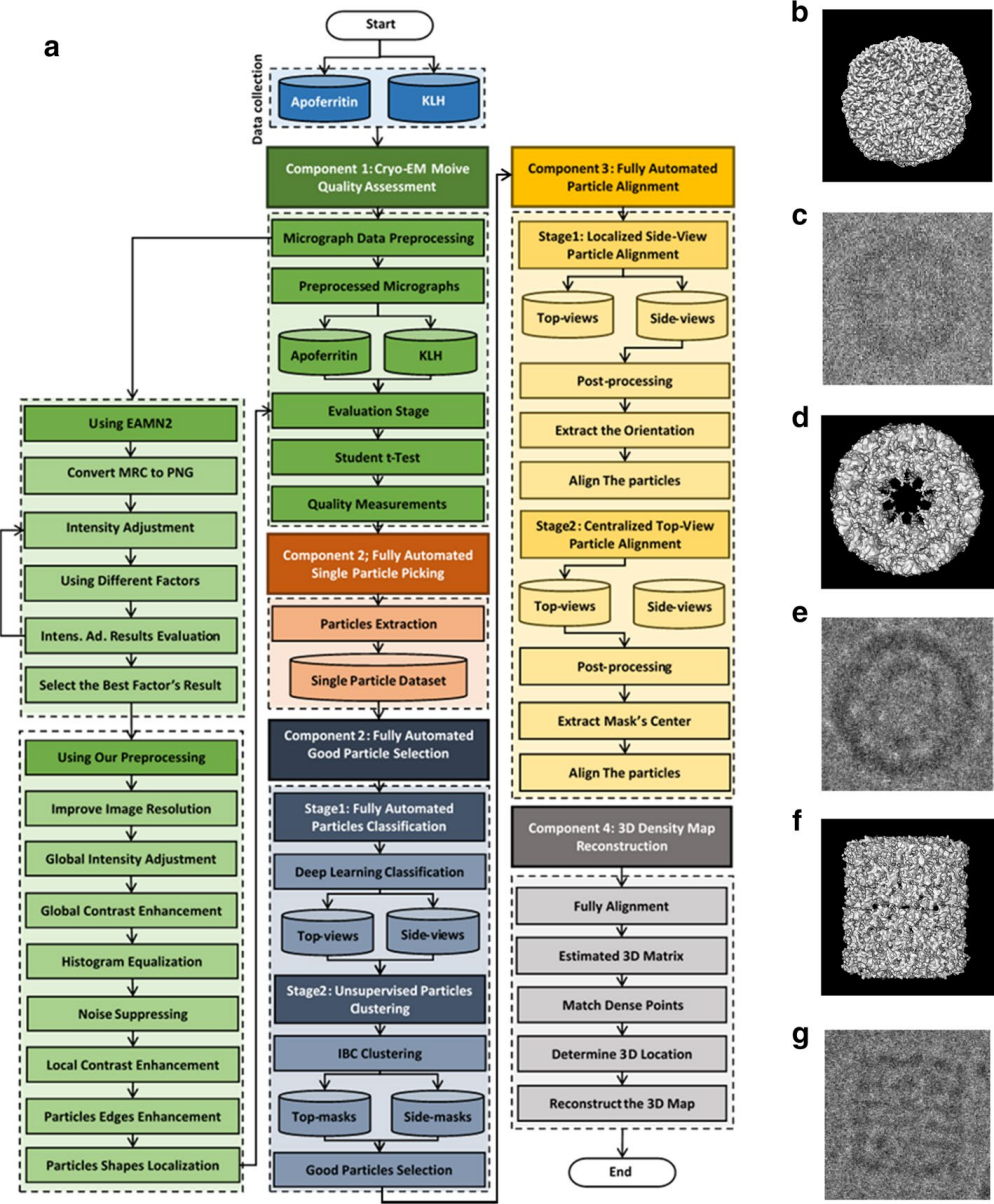
The general workflow of the Auto3DCryoMap. **a** The general workflow of the Auto3DCryoMap. The green part of the workflow shows the micrographs data preprocessing. The red part of the workflow shows the fully automated single-particle picking using DeepCryoPicker [10]. The blue part of the workflow shows the general flow of the good 2D single-particle selection using unsupervised learning and deep learning classification. The yellow part of the workflow shows the fully automated particle alignment. The gray part shows the 3D density map reconstruction using single-particle reconstruction. **b** Simulated top-view (circular) of the Apoferritin molecule shapes. **c** Apoferritin real-world top-view (circular) protein shape. **d** Simulated top-view (circular) of the KLH molecule shapes. **e** KLH real-world top-view (circular) protein shape. **f** Simulated side-view (square) KLH molecule shape. **g** KLH real-world side-view (circular) protein

We design Auto3DCryoMap—a fully automated 3D cryo-EM density map reconstruction method based on deep learning and unsupervised learning approaches. It is designed to reconstruct a 3D density map of a single protein from its cryo-EM image/micrograph data (see Fig. 1b, g) for examples from Apoferritin [11] and KLH [12] datasets). The workflow of the Auto3DCryoMap framework is shown in Fig. 1a. The workflow has five components described in detail as follows.

### Component 1: Micrograph pre-processing

In this component, a set of pre-processing steps that were proposed in our last three models AutoCryoPicker [13], SuperCryoEMPicker [14], and DeepCryoPicker [10] are used to improve the quality of the cryo-EM images and accommodate the low-SNR images. The preprocessing steps increase the particle’s intensity and group the pixels inside each particle to make it easier to be isolated.

### Component 2: Fully automated single particle picking

**Fig. 2:**
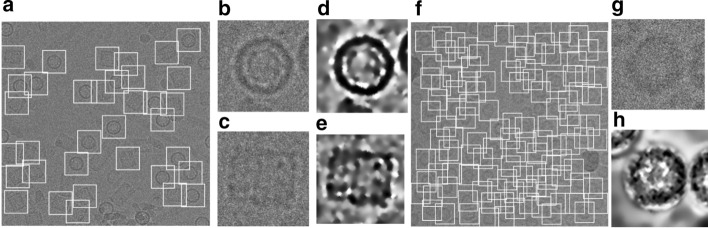
Fully Automated Particle Picking Results. **a**, **f** The particle picking results using different datasets (Apoferritin [11] and KLH [12]). **b**, **c** The original KLH particle picking results. **d**, **e** The preprocessed KLH particle picking results. **g** The original Apoferritin particle picking results. **h** The Apoferritin preprocessed particle picking results

The particles are first detected and picked using the DeepCryoPicker [10] and are then projected back onto their original ones to pick two versions of particles (original particle and the preprocessed one). Figure 2b, f show the whole micrograph particle picking results using different datasets (Apoferritin [11] and KLH [12] datasets). Figure 2b, c, g show the original versions of the picked particles while Fig. 2d, e, h show the preprocessed versions.

### Component 3: Fully automated perfect 2D particle selection

**Fig. 3:**
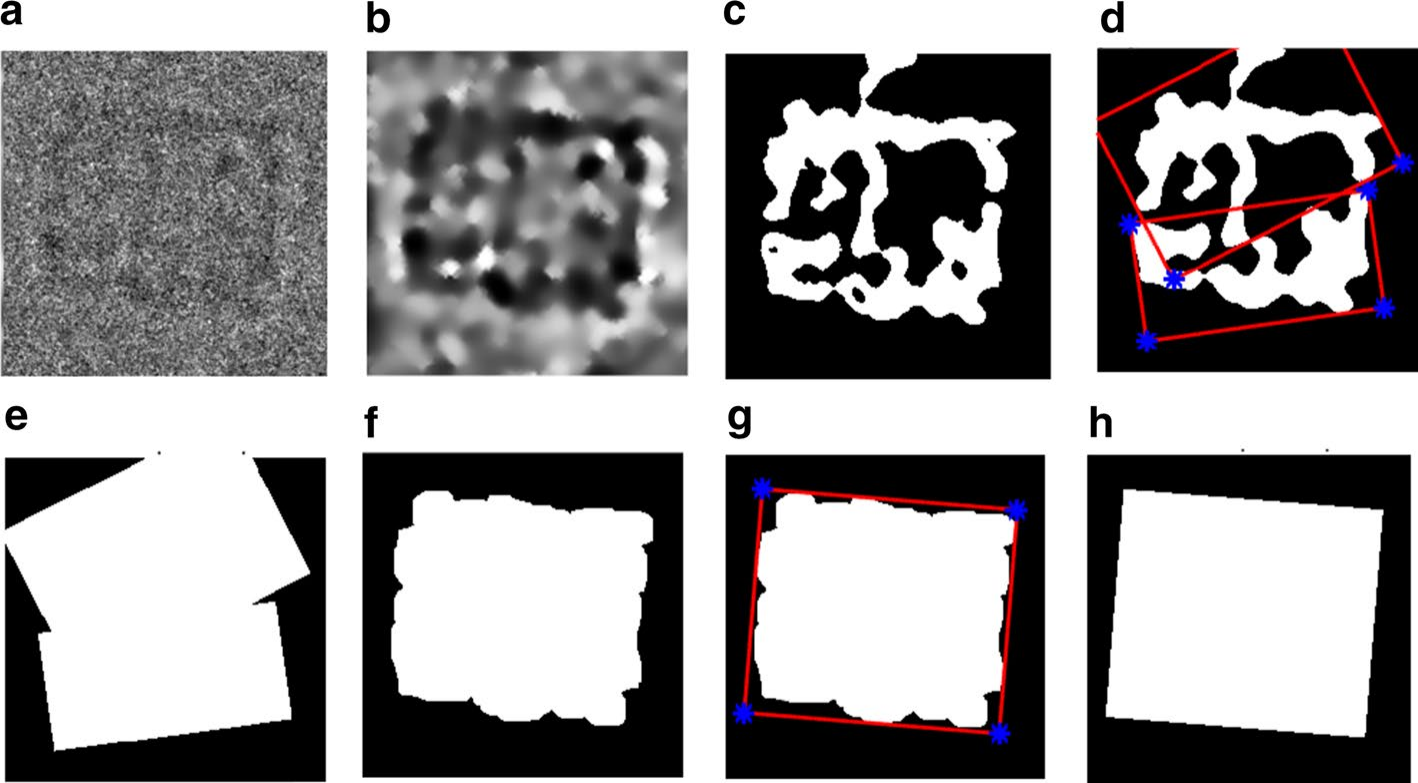
Perfect “good” 2D side-view particle selection-based fully automated perfect 2D binary mask generation. **a** Original side-view particle image that is picked by the DeepCryoPicker [10] using the KLH dataset [12]. **b** The preprocessed version of the original side-view particle. **c** Initial binary mask of the **b** using the IBC clustering algorithm in AutoCryoPicker [13]. **d** Feret diameter detection of **c**. **e** Initial 2D binary mask generation of **a**. **f** Postprocessing version of **c**. **g** Perfect Feret diameter detection using **f**. **h** Perfect binary mask generation

This component is designed to select good 2D particle images and generate a 2D particle mask for 2D particle alignment. This component is proposed in our last model DeepCryoPicker [10]. Each original particle image is evaluated and selected based on its particle’s mask using fully automated perfect 2D side-view particles selection algorithm (the details of Additional file 1: Algorithm S1). First, the original side-view particles are picked using the KLH dataset [12] (see an example in Fig. 3a). The preprocessed versions of the original side-view particle are used to generate initial binary masks using the intensity-based clustering algorithms (IBC) [13] (see an example in Fig. 3b, c). Then, the Feret diameter is applied on the initial binary masks to get the perfect side-view dimensions and generate perfect side-view binary masks (see Fig. 3d). It is noticed that the result is not quite accurate and the generated binary masks are not perfect (see Fig. 3e). For this reason, the initial binary masks are cleaned using fully automated 2D particle binary mask cleaning and post-processing algorithms (the details of Additional file 1: Algorithm S2). In this case, the Perfect Feret diameter detection produces perfect side-view binary masks (see Fig. 3g, h).

#### Step 1: Perfect “good” 2D side-view particle selection

This step is designed to select perfect 2D side-view particles (square shapes). It is based on using the individual binary mask of each particle that picked from the KLH [12] datasets as shown in Fig. 3c. First, a binary mask of each clustered particle image is cleaned by removing the small and irrelevant objects (Fig. 3f). The cleaned particle’s binary images contain almost only the square objects (side view particles). The connected components of each object are identified. Some artifact objects are removed. Finally, bounding boxes are drawn around each object, including a list of pixel locations using the Feret diameter measures approach [15] (see Fig. 3g). The details are described in Additional file 1: Algorithm S1. Also, the particle binary mask post-processing for particle image cleaning, small object, and irrelevant removal algorithm is shown in Additional file 1: Algorithm S2. An example of illustrating the input, intermediate results, and final output of the algorithm (perfect binary mask generation for the side-view particle) is shown in Fig. 3.

#### Step 2: Perfect “good” 2D top-view particle selection

**Fig. 4:**
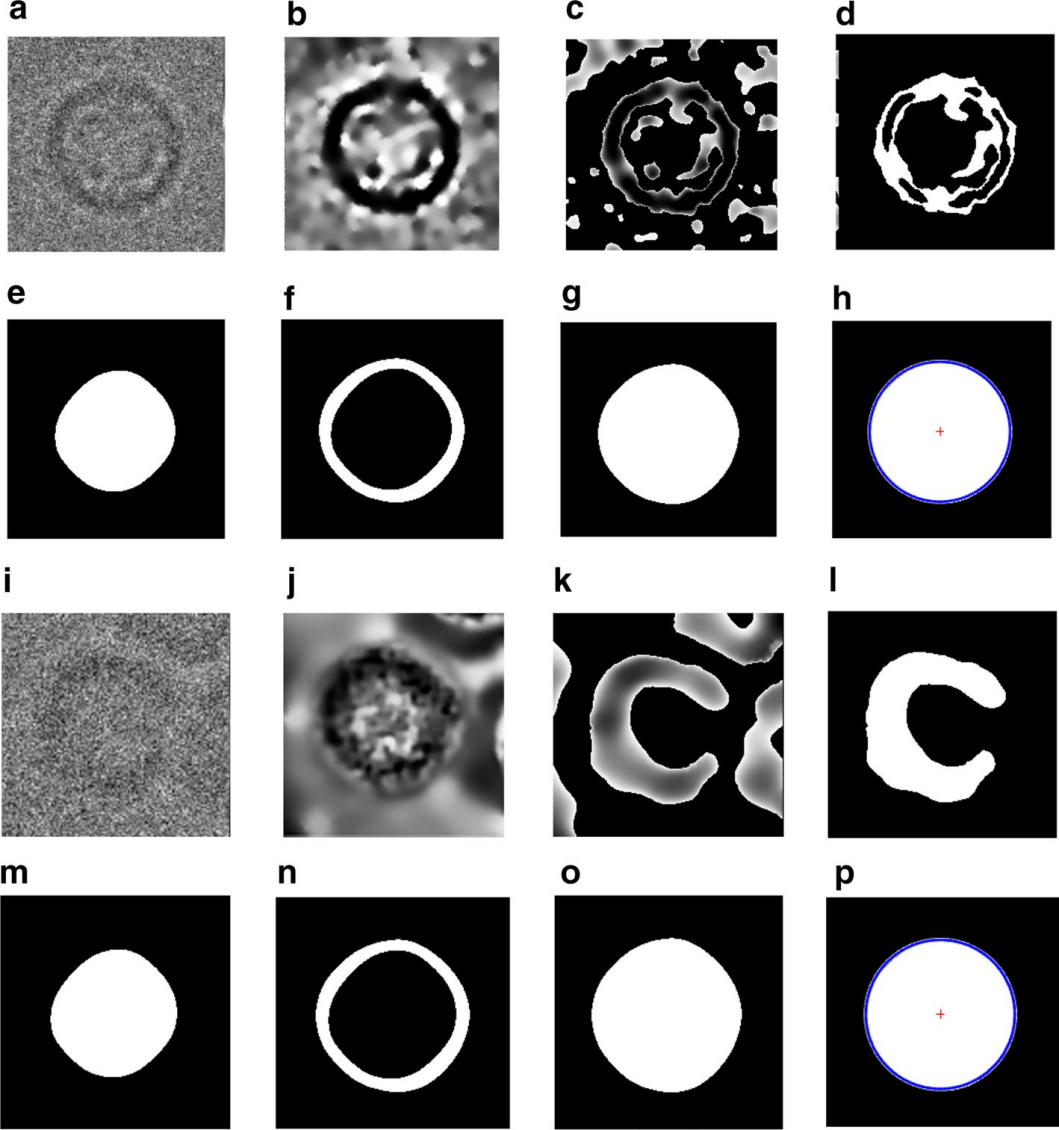
Perfect “good” 2D top-view particle selection-based fully automated perfect 2D binary mask generation. **a**, **i** Two original top-view particles that are fully automated picked using the DeepCryoPicker [10], Apoferritin [11], and KLH dataset [12]. **b**, **j** The preprocessed versions of the original top-view particles of **a**, **i** respectively. **c**, **k** The initial clustering results of the **b**, **j** using the IBC clustering algorithm in AutoCryoPicker [13]. **d**, **l** The cleaned circular clustered images of **c**, **k** respectively. **e**, **m** The outer circular mask extraction of the **d**, **l** respectively. **f**, **n** The inner circular mask of **e**, **m** respectively. **g**, **o** The filled circular binary masks of **f**, **n** respectively. **h**, **p** Perfect top-view binary mask generation of **g**, **o** respectively

This step is designed to select the top-view particles (circular shapes). It is based on using the individual binary mask of each particle (see Fig. 4c, k for two different top-view particles picked from different datasets (Apoferritin [11] and KLH [12] datasets). The details of the method are described in Additional file 1: Algorithm S3. An example of illustrating the input, intermediate results, and final output of the method (perfect binary mask generation for the top-view particle) is shown in Fig. 4. The preprocessed version of each particle (see Fig. 4d, j) is used to produce the initial clustering masks (see Fig. 4c, k) using the IBC clustering algorithm [13].Then, a cleaned binary mask of each particle image is produced by removing the small and irrelevant objects (Fig. 4d, l). The outer and inner circular mask are extracted (see Fig. 4e, f, m, n) to produce filled circular binary masks (see Fig. 4g, o. Finally, perfect top-view binary masks are generated using the center and the artificial dimeter of the modified CHT algorithm [13] (see Fig. 4h, p).

Once the particles are picked and selected, the perfect 2D mask for each particle is generated, the fully automated single-particle alignment is performed to perfectly align the particle images. This component consists of two stages: (1) Stage 1: fully automated side-view particle alignment; (2) Stage 2: full automated top-view (circular) particle alignment.

#### Step 3: Fully automated 2D side-view particle alignment

This step is designed to fully automated side-views particles. Particle alignment relies on placing the particle into a similar orientation [16]. Based on the relative plane of the two images, particles are shifted by $\left[ {x,y} \right]{ }$ or/and rotated by $\left( \varphi \right)$. Technically, image alignment needs to determine the correlation parameters $\left[ {x,y,\varphi } \right]$ to map the images perfectly [17–19]. Image registration aims to geometrically estimate, and match two images based on different viewpoints [20, 21].

Mathematically, the image registration is based on finding the best geometrical transformation that matches the same points on two images. Assume that $I_{F} \left( {x,y} \right)$ is the fixed or the reference image and $I_{M} \left( {x,y} \right)$ is the moving image (an image that needs to be aligned). The mathematical approach to estimate the geometrical transformation for the image registration $T\left( {x,y} \right)$ is based on Eq. (1) [22]:

1 $$T\left( {x,y} \right) = \left( {T_{1} \left( {x,y} \right),T_{2} \left( {x,y} \right)} \right)$$

Such that using the estimated geometrical transformation $T\left( {x,y} \right)$ to register the moving image $I_{M} \left( {x,y} \right)$ to produce the close image $I_{c} \left( {x,y} \right)$ using the following Eq. (2) [23]:

2 $$I_{c} \left( {x,y} \right) = I_{M} \left( {T\left( {x,y} \right){ }} \right) \approx I_{R} \left( {x,y} \right)$$

Thus, the image registration can be formatted as a maximization problem-based optimizer function that is shown in Eq. (3) [23]:

3 $$T_{opt} = \mathop {{\text{argmax}}}\limits_{{T \in {\mathcal{T}}}} S\left( {I_{R} ,I_{M} \left( T \right)} \right)$$

where $T_{opt}$ denotes as the optimal geometrical transformation for $I_{R} \left( {x,y} \right)$ and $I_{M} \left( {x,y} \right)$ matching based on the selected metric of the measurement similarity $\left( S \right)$ among the specific transformation $\left( {\mathcal{T}} \right)$. Finally, the geometrical transformation $T\left( {x,y} \right)$ follows the 2D parametric model to estimate the continuous bivariate function to estimate the certain regularity conditions [20, 21] based on the following Eq. (4) [23, 24]:

4 $$\left\{ {\begin{array}{*{20}c} {T_{1} \left( {x,y} \right) = \alpha \left( {x\cos \Delta \phi + y\sin \Delta \phi } \right) + \Delta x} \\ {T_{2} \left( {x,y} \right) = \alpha \left( {x\cos \Delta \phi + y\sin \Delta \phi } \right) + \Delta y} \\ \end{array} } \right.$$

where $\left( {\Delta x,\Delta y, \Delta \phi } \right)$ are the three geometrical (motional) parameters and $\alpha$ is the geometrical scaling parameter.

Intensity-based image registration is an image registration process which is based on the intensity image similarity to define the 2D geometrical transformation for minimizing or maximizing the similarity metric [25]. It is based on the estimation of the internal geometrical transformation matrix $T\left( {x,y} \right)$ after applying the image transformation (bilinear interpolation [26]) on the two images $I_{F} \left( {x,y} \right)$ and $I_{M} \left( {x,y} \right)$. The idea from applying the bilinear interpolation on both images is that the bilinear interpolation [26] is one of the resampling techniques (image scaling) on the computer vision and the image processing which transform that image to a specific transformation $\left( {\mathcal{T}} \right)$.Then, the measurement similarity $\left( S \right)$ of the transformed images is used to estimate the geometrical transformation $T\left( {x,y} \right)$ using a mean square error (MSE) as a confirmation metric that is used to measure the similar $\left( S \right)$ between the two transformed images $I_{R} \left( {x,y} \right)$ and $I_{M} \left( {x,y} \right)$ based on the following Eq. (5) [27, 28]:

5 $$MSE = \frac{1}{m \times n}\mathop \sum \limits_{x = 0}^{m - 1} \mathop \sum \limits_{y = 0}^{n - 1} \left[ {I_{c} \left( {x,y} \right) - I_{F} (x,j{\text{y}}} \right]^{2}$$

Then, the regular step gradient descent optimization [29] is used to estimate the optimizer parameters. The regular step gradient descent optimization [29] uses to adjust the geometrical transformation parameters by following the gradient of the image similarity metric in the direction of the extrema [30]. Equation (9) shows the typical form of gradient descent optimization used to estimate the image registration optimizer [30].

6 $$X_{\eta + 1} = X_{\eta } - \gamma \nabla F\left( {X_{\eta } } \right)$$

where $\gamma \nabla F\left( {X_{\eta } } \right)$ gradient factor that is a subtraction from $X_{0}$ to make it move to the global minimum (stop condition), and $X_{0}$ is the local minimum of the main function $F$ which is in our case the similarity metric $\left( S \right)$. Finally, the image registration function maps each point in the moving image $I_{M} \left( {x,y} \right)$ into the corresponding point in the reference image $I_{R} \left( {x,y} \right)$ based on the estimated correlation parameters $\left[ {x,y,\varphi } \right]$ from the similarity metric and optimizer functions.

Typically, a different geometrical transformation can be used to register the two images such as translation, scaling, rotation, and affine transformation as is shown in Eqs. (7), (8), and (9) [31].

7 $$P = T + P^{\prime}$$

8 $$P = \left[ {\begin{array}{*{20}c} x \\ y \\ \end{array} } \right],{ }P^{\prime} = \left[ {\begin{array}{*{20}c} {x^{\prime}} \\ {y^{\prime}} \\ \end{array} } \right],{ }T = \left[ {\begin{array}{*{20}c} {d_{x} } \\ {d_{y} } \\ \end{array} } \right]$$

9 $$x^{\prime} = x + d_{x} { }\;{\text{and}}\;y^{\prime} = y + d_{y}$$

Using the scaling transformation, the new point $P\left( {x,y} \right)$ is scaled along $x$ and $y$ axis to a new point $P\left( {x^{\prime},y^{\prime}} \right)$ (see Eqs. (10) and (11)) by multiplying $x$ and $y$ by the scaling factors $S_{x}$ and $S_{y}$ (see Eq. (12)) [32]:

10 $$P = S \times P^{\prime}$$

11 $$P = \left[ {\begin{array}{*{20}c} {x^{\prime}} \\ {y^{\prime}} \\ \end{array} } \right] = \left[ {\begin{array}{*{20}c} {S_{x} } & 0 \\ 0 & {S_{y} } \\ \end{array} } \right] \times \left[ {\begin{array}{*{20}c} x \\ y \\ \end{array} } \right]$$

12 $$x^{\prime} = x + d_{x} { }and{ }y^{\prime} = y + d_{y}$$

By using the rotational transformation, the new point $P\left( {x,y} \right)$ is rotated around the origin to a new point $P\left( {x^{\prime},y^{\prime}} \right)$ by an angle $\theta$ (see Eqs. (13), (14), and (15)) [33]:

13 $$x^{\prime} = x \times \cos \theta - y \times \sin \theta$$

14 $$y^{\prime} = x \times \sin \theta + { }y \times \cos \theta$$

15 $$P = \left[ {\begin{array}{*{20}c} {x^{\prime}} \\ {y^{\prime}} \\ \end{array} } \right] = \left[ {\begin{array}{*{20}c} {\cos \theta } & {\sin \theta } \\ {\sin \theta } & {\cos \theta } \\ \end{array} } \right] \times \left[ {\begin{array}{*{20}c} x \\ y \\ \end{array} } \right]$$

**Fig. 5:**
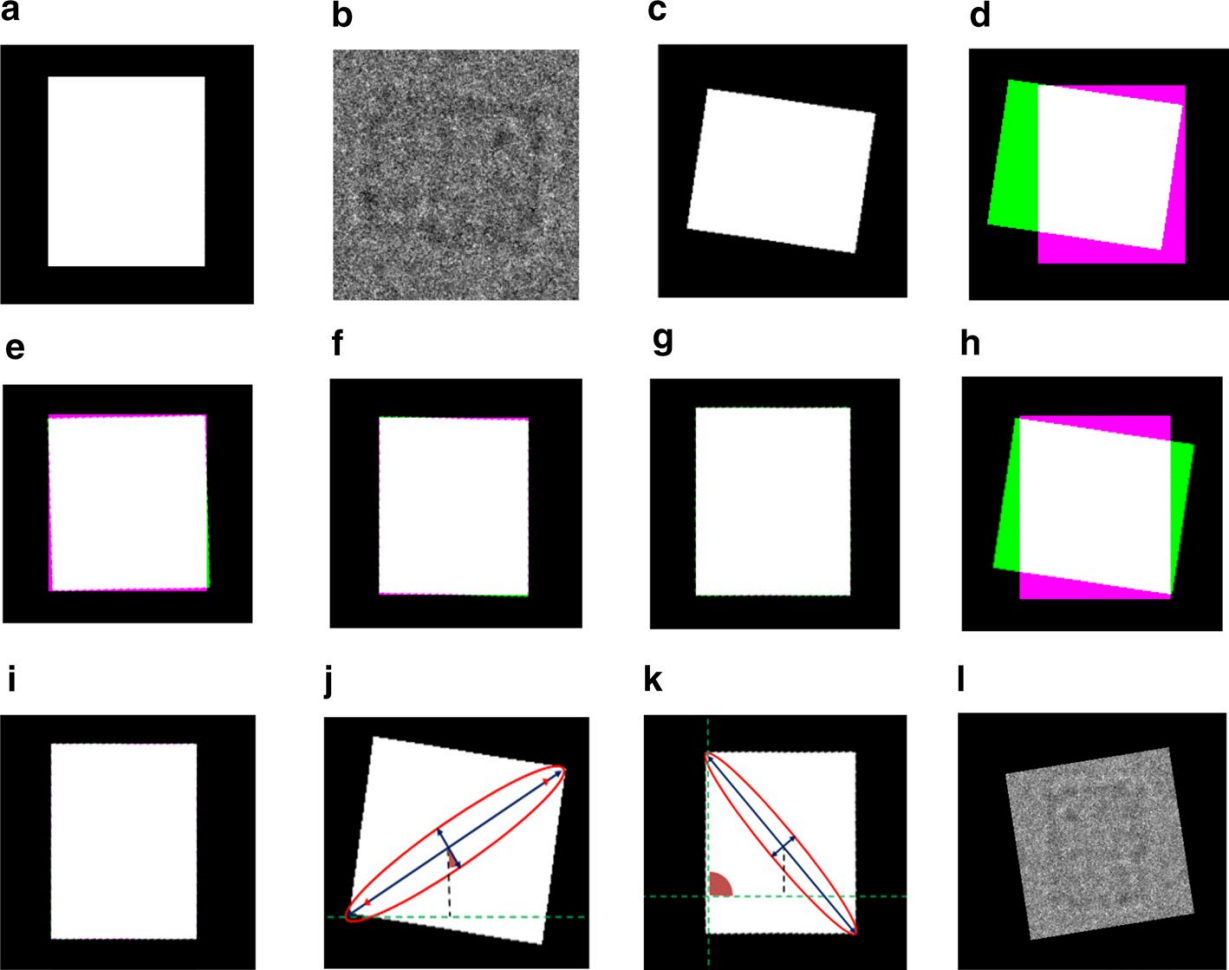
Fully automated 2D side-view particle alignment using KLH dataset [12]. **a** Generated frontal view reference image using average binary particle object sizes. **b** Original side-view particle image (moving). **c** Perfect generated binary mask of **b**. **d** Unalignment images projection (references **a** and moving **c**). **e** Default image alignment (initial registration). **f** Optimizer adjustment and metric configuration-based image registration. **g** Image registration-based on increasing the maximum iteration number. **h** Image registration-based optimization and rigid transformation. **i** Image remigration using affine transform. **j** Original binary mask particle’s orientation. **k** Aligned binary mask particle’s orientation. **l** Final particle alignment result

In some cases, the translation and rotation are not enough. However, the scaling is necessary to correct the transformation of the point in the $I_{M} \left( {x,y} \right)$. Therefore, the affine transformation scales the translation and rotational points (see Eq. (16)) based on using the two-dimensional shear transformation as is shown in Eq. (17) [34]:

16 $$T_{Scale} \left( {x,y} \right) = \left[ {\begin{array}{*{20}c} {x^{\prime}} \\ {y^{\prime}} \\ \end{array} } \right] = S \times \left[ {\begin{array}{*{20}c} x \\ y \\ \end{array} } \right]$$

17 $$SH_{x} = \left[ {\begin{array}{*{20}c} 1 & a & 0 \\ 0 & 1 & 0 \\ 0 & 0 & 1 \\ \end{array} } \right]\;{\text{and}}\;SH_{y} = \left[ {\begin{array}{*{20}c} 1 & 0 & 0 \\ b & 1 & 0 \\ 0 & 0 & 1 \\ \end{array} } \right]$$

where $a$ and $b$ are the proportionality constants along axis $x$ and $y$, respectively [36]. Since in the second stage of the third component of our Auto3DCryoMap framework “fully automated perfect 2D particles-selection,” which is “stage 2: fully automated 2D particle mask generation based unsupervised learning approach”, perfect binary masks are generated, we propose a fully automated approach for perfect side-view particle alignment-based automatic intensity-based Image registration using the perfect generated particle binary masks. In terms of the fully automated approach, we use the binary masks instead of the original particle images for two reasons. The first one is it is easier to automatically generate a reference image than manually select one. Second, it is easier to find the correlation points (corresponding corners) in the generated mask than the original particle image since the signal-to-noise ratio is very low-intensity value. The main steps to do the fully automated particle alignment-based intensity image registration using the perfect generated binary masks are as follows: First, we calculate the average binary particle object sizes and generate an artifice frontal view reference image (side-view) particle as is shown in Fig. 5a. Second, for each particle image, we use the original particle image and the generated binary mask as is shown in Fig. 5b, c. Then, we use intensity-based automated image registration to align the perfect generated binary mask of each particle based on the generated reference binary mask (frontal view) using deferent geometrical transformation (see Fig. 5e–i). After the perfect alignment is done, we extract the angles of both the aligned object and the original mask $\theta_{orginal}$ and $\theta_{aligned}$ which is the angle between the x-axis and the major axis of the object that has the same second-moments as the region (see Fig. 5j, k). Then, we extract the orientation angle $\theta_{orentaion}$ based the on the difference between the aligned angle and the original angle. Finally, we use the orientation angle $\theta_{orentaion}$ to rotate the original particle image as is shown in Fig. 5l.

**Fig. 6:**
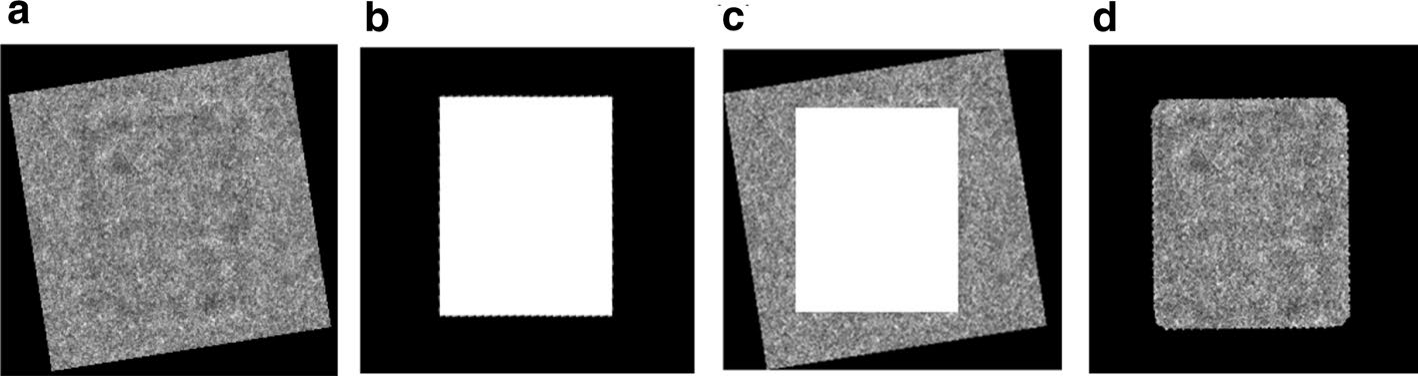
Fully automated localized 2D top-view particle alignment using the generated 2D binary mask. **a** Original aligned particle image. **b** Perfect 2D particle binary mask of the original image **a**. **c** Perfect 2D particle binary mask projection with its original particle image. **d** Localized 2D top-view aligned particle image

To improve the SNR, class averaging is used over fewer particles to conduct the resolution. The researchers used to manually pick particles and do the 2D image averaging to remove some false positive particles from the whole data. Instead of using the extraction of the individual particle from micrograph background that is proposed in RELION [8], which uses a manually user-defined radius, a circle (normalization procedure), to extract each particle image in a background area (outside the circle) and a particle area (inside the same circle) [35] and do the image averaging, we proposed a localized image averaging approach. The localized particle image is generated based on using the binary mask for each particle as is shown in Fig. 6. In this case, the original aligned particle image (see Fig. 6a) is multiplied by the perfect 2D aligned mask (see Fig. 6b) to generate the perfect 2D localized particle images (see Fig. 6d). The fully automated side-view particle alignment-based intensity image registration and particle masks are described in Additional file 1: Algorithm S4 and the whole framework of the fully automated side-view particle alignment-based intensity image registration is illustrated in Additional file 1: Figure S1.

#### Step 4: Fully automated 2D top-view particle alignment

**Fig. 7:**
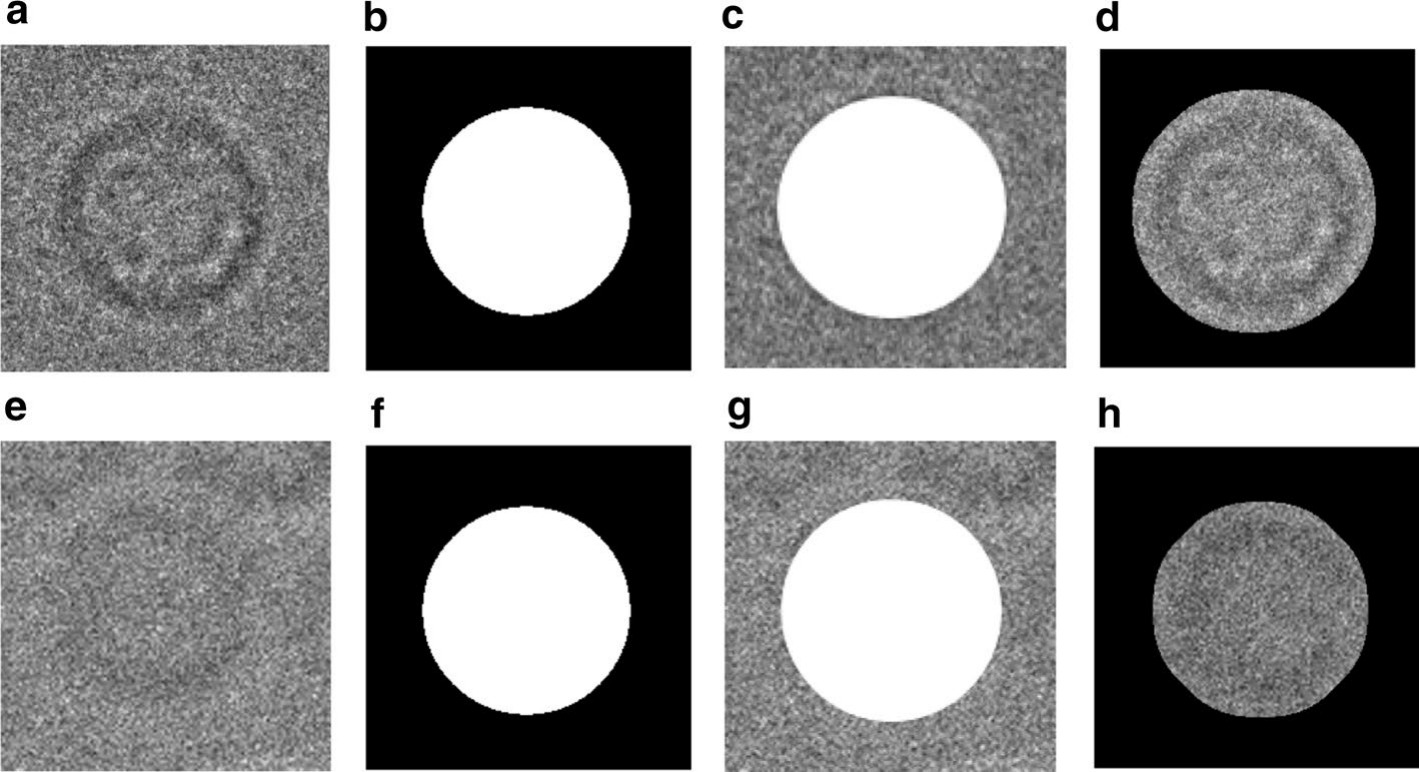
Fully automated 2D top-view particle image alignment using the generated 2D binary mask. **a**, **e** Original top-view particle from Apoferritin [11] and KLH [12] datasets. **b**, **f** Perfect 2D top-view particle binary mask of **a**, **b** respectively using the modified CHT algorithm [13] and the IBC [13]. **c**, **g** Perfect 2D top-view particles projection results. **d**, **h** Centralized top-view of the localized particle alignment result

This step is designed to align the other common type of the particle images top-view (circular shapes). The top-view particle images are aligned based on centralizing all particles together on the same point which is a common way to align the circular particle. Since one particle (original form) might be heavy noisy compared to another one, it is very hard to find the same center point to centralize them. Also, circular particles can be miss centered, especially those that have a hollow in the particle ring. To come up with a perfect fully alignment approach, we propose a localized top-view centralization approach for top-view (circular) particle alignment. First, we used the localization approach that is applied to the side-view particle images to produce localized top-view particle images (see Fig. 7d).

**Fig. 8:**
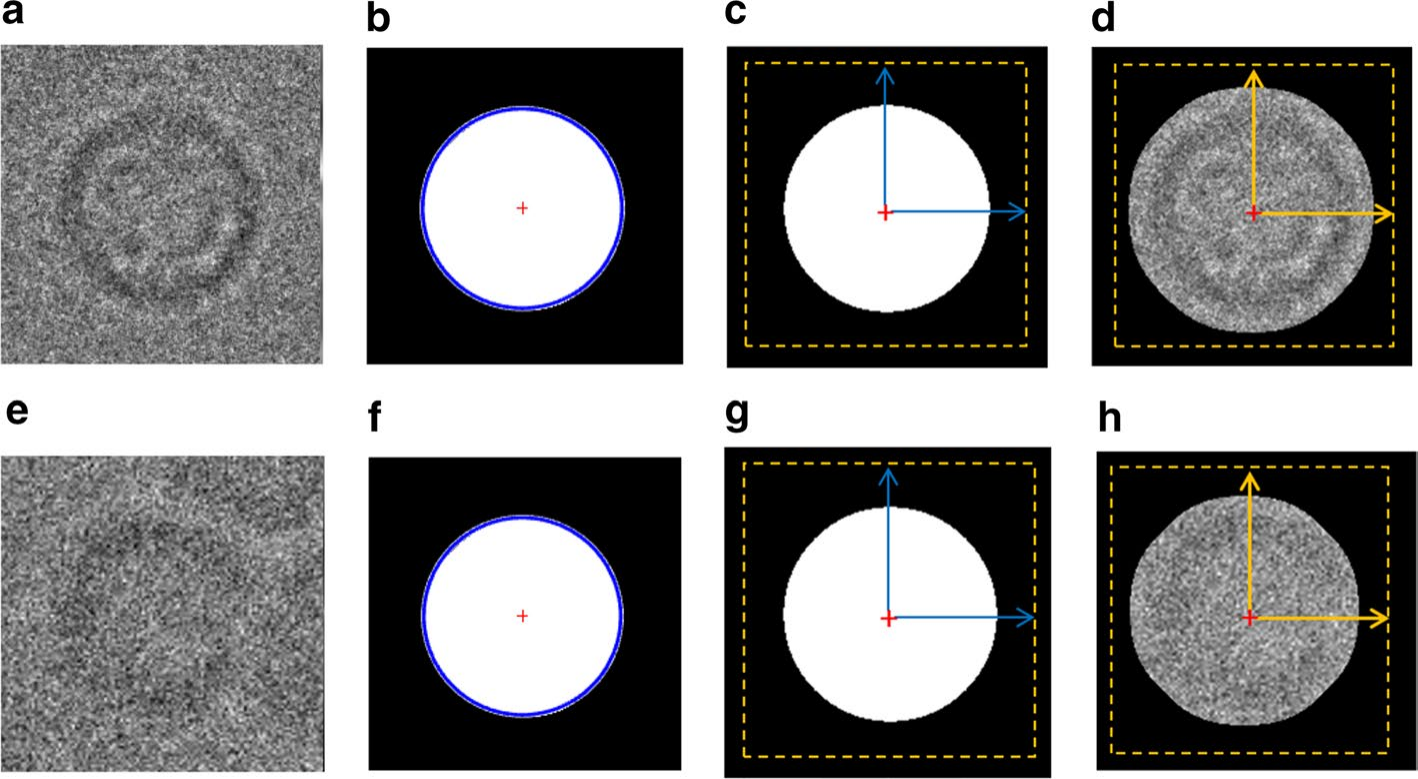
Fully automated perfect 2D top-view particle alignment-based particle’s centralization approach. **a**, **e** Original top-view particle images from Apoferritin [11] and KLH [12] dataset. **b**, **f** Center point extraction using the modified CHT algorithm [13] and the generated perfect binary masks for **a**, **b** respectively. **c**, **g** Centralized top-view particle binary mask alignment result. **d**, **h** Perfect centralized top-view of the localized particle alignment result

Second, it is more accurate to find the same center point (center of the binary circle) and the ring hollow is not a part of the centralization issue anymore. The center of the circular object (binary) is defined as an average of all points in the circular shape [36]. Suppose that that circular shape consists of $n$ points $x_{1} ,x_{2} \ldots ,x_{n} { }$(white pixels) as is shown in Fig. 8a, e. The centroid (center) of the circular white (binary) object is defined based on the Eq. (18) [36]:

18 $$centerid = \frac{1}{n}\mathop \sum \limits_{i = 1}^{n} x_{i}$$

In our case, we use the modified CHT [13] to extract the exact center point of each particle’s mask as is shown in Fig. 8b, f. Then, from the extracted center point we draw a new candidate box the takes the same dimension ($$x_{width} ,x_{hights} )$$. New bounding boxes are drawn around each top-view region (rectangle region area) after increasing each object center $$\left( {x,y} \right)$$ using the same factor value and calculate the bounding boxes dimensions ($$x_{width} ,x_{hights} )$$ (see Fig. 8c, g). This approach allows the particles that have hollows (rings) to be accurately aligned based on the same particle mask extracted center (see Fig. 8d, h). Centralized based particle alignment-based perfect binary mask generation allows the particles to be placed (aligned) in the same point (center) which will help the 3D map reconstruction to overlap the particles in which they need to be aligned, shifted in the plane.

**Fig. 9:**
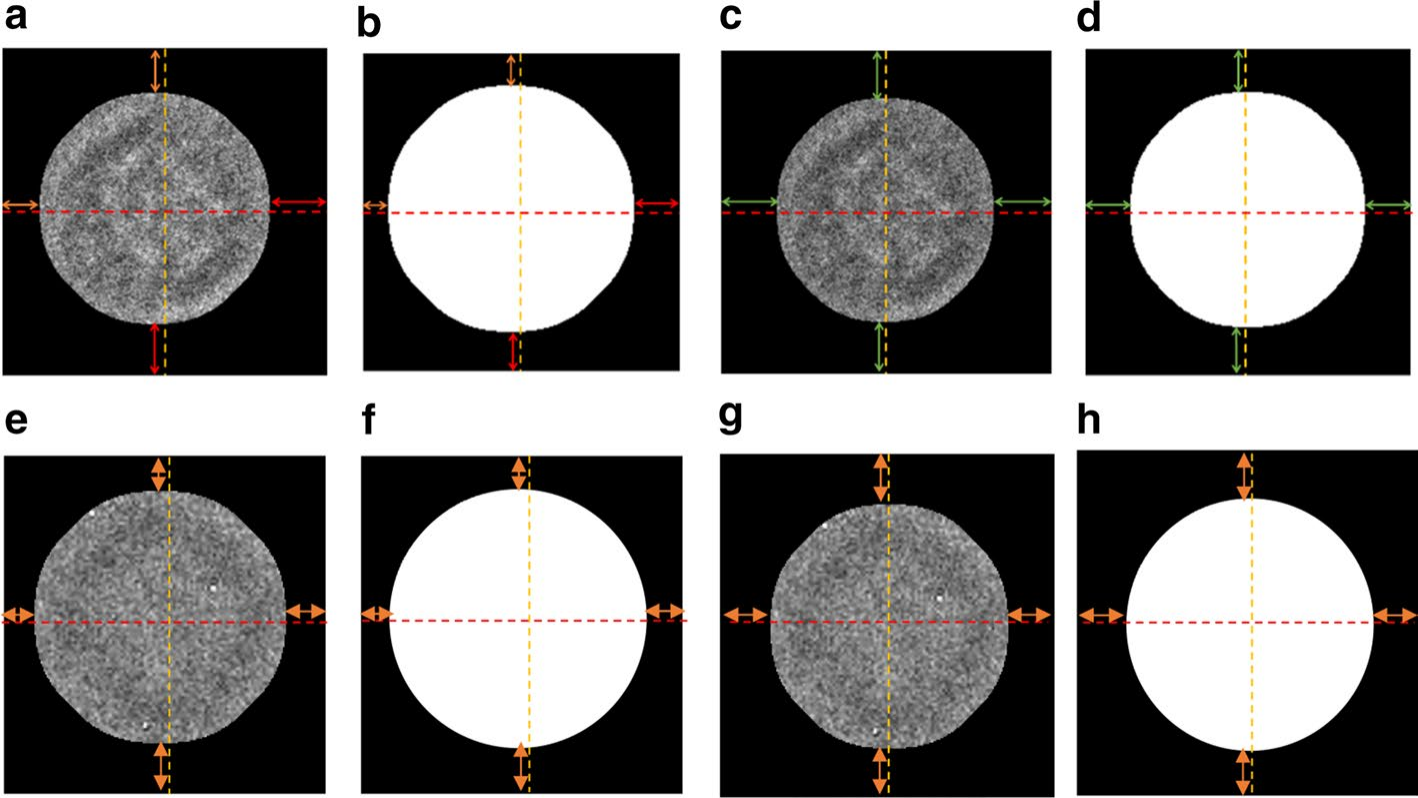
Comparing the localized 2D particle image results before and after the perfect top-view particle centralized based particle image alignment. **a**, **e** Localized 2D top-view particle images from the KLH [12] and Apoferritin [11] datasets before the centralized particle image alignment. **b**, **f** Localized 2D top-view particle binary masks of **a**, **e** before the centralized particle image alignment. **c**, **g** Localized 2D top-view particle images of **a**, **e** after centralized based particle image alignment. (d, h) Localized 2D top-view particle masks of **a**, **e** after centralized based particle image alignment

The extracted correlation point (center) that is determined based on the extraction of the center of the perfect binary mask (see Fig. 9b) allows the particle to be shifted along with the fixed center point (see Fig. 9c). In this case, all the particles are cross correlated to each other and shifted as necessary to the same center point. Figure 9 shows an example of two top-view particles from two different datasets before and after the centralized alignment-based perfect generated binary mask. The particle image is shifted as best as possible to be centrally aligned. The fully automated approach for a centralized top-view particle alignment-based particle mask is shown in Additional file 1: Algorithm S5 and illustrated in Additional file 1: Figure S2.

### Component 4: 3D density map reconstruction

The basic idea of reconstructing the 3D density map based cryo-EM is to project the density depth of several thousands of 2D cryo-EM particles. The Fourier coefficient-based Fourier Transformation (FT) [37] is used to represent the 2D particles in another space (Fourier space), in which the structure of each 2D particle is represented in Fourier coefficients. In this case, the Fourier synthesis is used to reconstruct the 3D density map through its 3D Fourier transformation based on the direction of the projection [7, 38, 39]. This approach requires a huge number of 2D particles to build such significant Fourier coefficients that represent the particle object structure. To come up with the same descent 3D density map using a smaller amount of 2D particle images, we propose a new localized approach for 3D density map reconstruction based on the 2D shape appearance of the real object. Localized based 3D density map reconstruction approach bases on extracting the structural information-based particle object from every two 2D particle images [39].

Structural based motion information is a process that estimates the 3D structure (3D matrix) from a set of 2D images [40]. Different steps are implemented in this component to achieve the 3D density map reconstruction-based particle structural motion information. First, match the sparse set of points between every two 2D particle images based on perfect 2D image alignment. Second, estimate the fundamental matrix (3D matrix). Third, track a dense set of points between the two images that illustrate the estimated structure of the object (particle in the 3D). Then, determine the 3D locations of the matched points using triangulation. Finally, recover the actual 3D map based metric reconstruction. The 3D density map, in this case, is building based on only the first two particle images. In the end, the average of all localized 3D density map represents the final 3D density map. The whole framework of the localized 3D density map reconstruction is illustrated in Additional file 1: Figure S3.

#### Step 1: Perfect 2D particle alignment

**Fig. 10:**
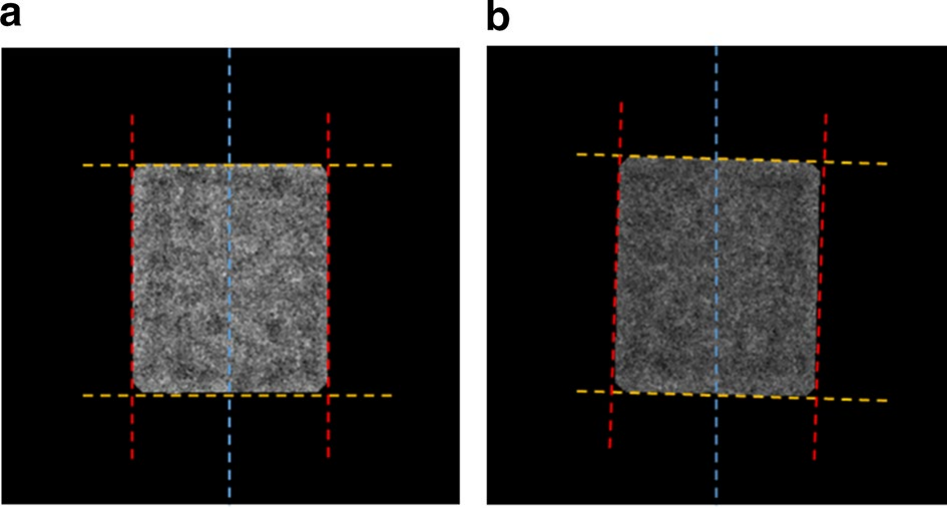
Localized 2D side-view particle image before the perfect 2D side-view particle alignment. **a**, **b** Two localized 2D particle images are not perfectly aligned

In terms of matching a sparse set of points between the two 2D particle images, there are multiple ways of finding point correspondences between two 2D particle images by detecting corners in the first image and tracking them into the second image. In side-view protein particle shapes, we discover that some cases our final localized 2D particle images are not aligned perfectly which causes the miss-tracking of the detected points (see Fig. 10a, b).

**Fig. 11:**
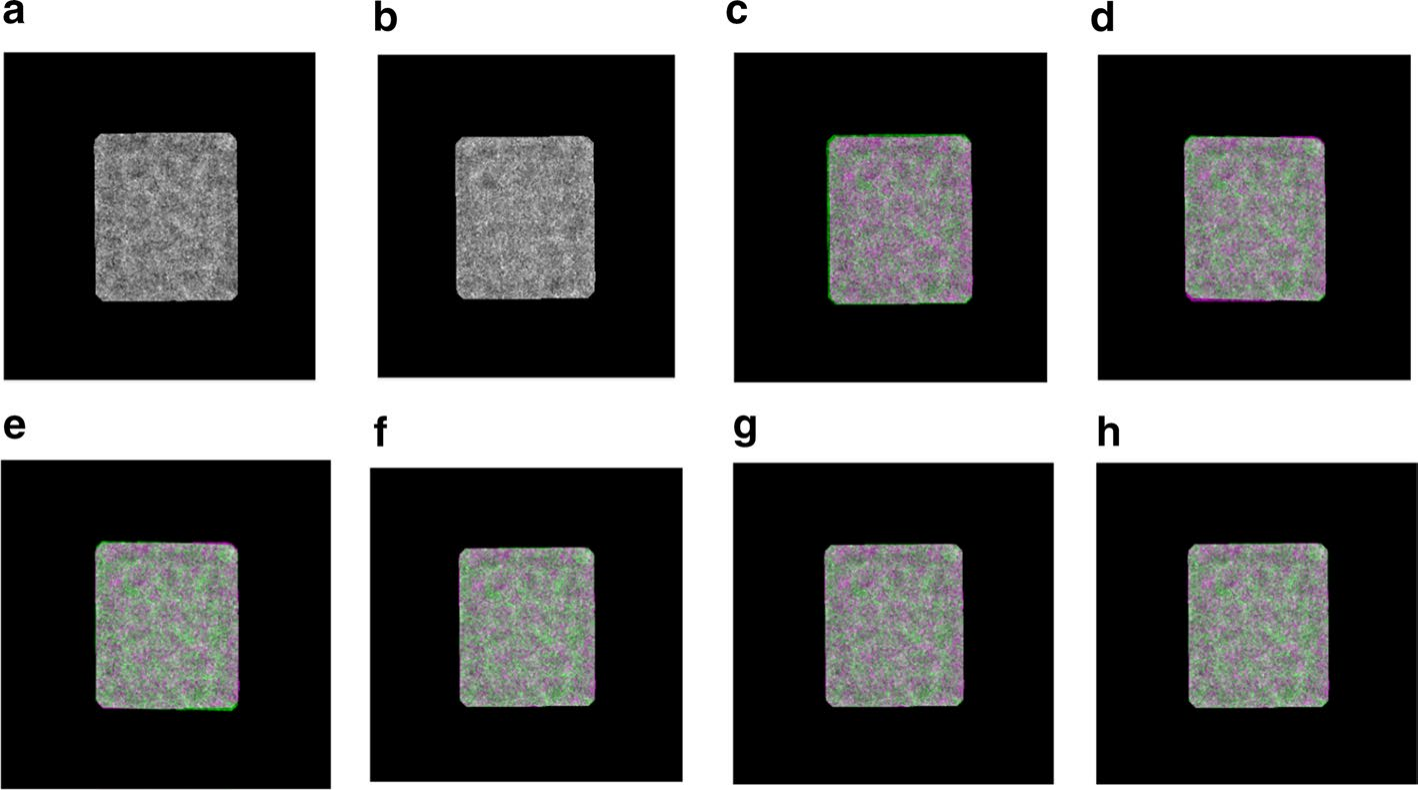
Fully automated perfect 2D side-view particle alignment results using the KLH dataset [12] and the intensity-based image registration. **a**, **b** Two localized aligned particle images that are not perfectly aligned. **c** Two-particle image projection (**a**, **b**). **d** Default image alignment (initial registration). **e** Optimizer adjustment and metric configuration-based image registration. **f** Image registration based increasing the maximum iteration number. **g** Image registration-based optimization and rigid transformation. **h** Image remigration using affine transform

To solve this issue, we use the same fully automated side-view particle alignment algorithm directly on the aligned particle images. Different transformation functions are used to perfectly align the two-particle images such as default alignment (initial registration) using affine transformation-based images scaling, rotation, and (possibly) shear (see Fig. 11d). We can notice that the default image alignment (initial registration) is very good. Thus, there are still some poor regions that are not perfectly aligned. To improve the image alignment, we use the optimizer adjustment and metric configuration properties which control the initial step length (size) that is used to adjust the parameter space to refine the geometrical transformation (see Fig. 11e). By increasing the maximum iteration number during the image registration process, that allows the image registration (alignment) to run longer and potentially to find significant registration results (see Fig. 11f). Image registration-based optimization works better than the initial registration. For this reason, we can improve the image alignment (registration) by starting with more complicated transformation such as ‘rigid’ [41] than the transformation result uses as an initial registration model by using the affine transform (see Fig. 11g). Another option is that the initial geometrical transformation is used to refine the image registration by using the affine transform with the similarity model. In this case, the refine model estimates the image registration result by including the shear transformation (see Fig. 11h).

#### Step 2: Extract and match set of sparse points

**Fig. 12:**
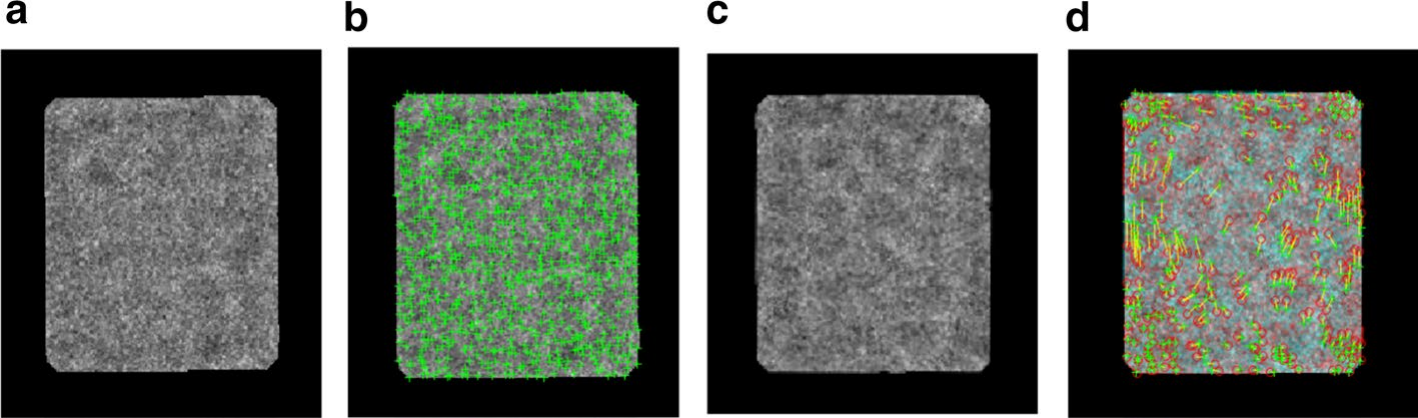
Sparse points matching and extraction results. **a** First tested particle image. **b** Features (corners) extraction using minimum eigenvalue algorithm [42]. **c** The second tested particle image. **d** Correlation points detection and tracking using Kanade-Lucas-Tomasi (KLT), feature-tracking algorithm [43–45]

After perfectly aligning all the particle images, the correlation points that will be extracted in this step will be accurately tracked because the interesting points are on the space. There are many ways to find the correlation (corresponding) points between two-particle images. To extract the corresponding points, the first particle image is used as a reference image and detects the corner points (features) using the minimum eigenvalue algorithm developed by Shi and Tomasi [42] and MATLAB function ‘detectMinEigenFeatures’ (see Fig. 12b). Then, the same extracted features (detected points) are tracked on the second image using the Kanade–Lucas–Tomasi (KLT), feature-tracking algorithm [43–45] and MATLAB function “PointTracker” (see Fig. 12d).

#### Step 3: 3D fundamental matrix estimation

The fundamental matrix is the estimated 3D matrix that relates to the corresponding points in two images [46–50]. The normalized eight-point algorithm [51] is used to estimate the 3D matrix based on using a list of corresponding points in every two-particle image. The fundamental matrix is specified based on the following Eq. (19) [51]:

19 $$\left[ {P_{2} } \right] \times 3D_{Fundematal\, Matrix} \times \left[ {P_{1} } \right] = 0$$

where $P_{1}$ is the point in the points list of the first image (list1) that is corresponding to $P_{2}$ which is the point in the point list of the second image (list2). The $3D_{{Fundematal\,Matrix}}$ estimates the outlier's points based on using a random sample consensus algorithm (RANSAC) [48]. To compute and estimate the $3D_{{Fundematal\,Matrix}}$ different steps are implemented. First, the $3D_{{Fundematal\,Matrix}}$ is initialized by producing a $3 \times 3$ matrix of zeros $F_{initail}$. Second, a loop counter, which iterated the whole process based on the specified number of trails, $N{ }$ is initialized. Each trail represents the estimated outlier points in the 3D matrix. For each iteration, 8 Paris points are randomly selected from each point list in the two images (corresponding points) in list1 and list2. Then, use the selected 8 points to compute the fitness function $f$ of the 3D fundamental matrix $F$ by using the normalized 8-point algorithm [51] based on the following Eq. (20) [51]:

20 $$\left( {y^{\prime}} \right)^{T} Fy = 0$$

where $y^{\prime}$ and $y$ are the corresponding selected points from list1 and list2 and $F$ is the estimated 3D matrix, which can be similarly written as Eq. (21) [51]:

21 $$f^{T} Y = 0$$

where $f$ is denoted as the reshape version of $3D_{{Fundematal\,Matrix}}$ $F$. After fitness function $f$ is computed based on the corresponding points in the two images, if the fitness function $f$ is better than the 3D fundamental matrix $F$, the 3D fundamental matrix $F$ is replaced with the fitness function $f$. Then, the random number of trails $N$ for every iteration is updated based on the RANSAC algorithm using Eq. (22) [51]:

22 $$N = \min \left( {N,\frac{{\log \left( {1 - p} \right)}}{{\log \left( {1 - r^{8} } \right)}}} \right)$$

where $p$ is denoted as the selected confidence parameters, and $r$ is calculated based on the Eq. (23) [51]:

23 $$\mathop \sum \limits_{i}^{N} \frac{{{\text{sgn}} \left( {du_{i} v_{i} ,t} \right)}}{N}$$

where ${\text{sgn}} \left( {du_{i} v_{i} ,t} \right)$ is the distance function that follows the following Eq. (24) [51]:

24 $${\text{sgn}} \left( {a,b} \right)\left\{ {\begin{array}{*{20}l} {1\quad if\;a \le b } \hfill \\ {0\quad otherwise} \hfill \\ \end{array} } \right.$$

Two different types of distance (algebraic and Sampson) are used to measure the distance of pair points as Eqs. (25) and (26) show respectively [51]:

25 $$d\left( {u_{i} ,v_{i} } \right) = \left( {v_{i} Fu_{i}^{T} } \right)^{2}$$

26 $$d\left( {u_{i} ,v_{i} } \right) = \left( {v_{i} Fu_{i}^{T} } \right)^{2} = \left[ {\frac{1}{{\left( {u_{i} Fu_{i}^{T} } \right)_{1}^{2} + \left( {v_{i} Fu_{i}^{T} } \right)_{2}^{2} }} + \frac{1}{{\left( {v_{i} Fu_{i}^{T} } \right)_{1}^{2} + \left( {v_{i} Fu_{i}^{T} } \right)_{2}^{2} }}} \right]$$

where $i$ is denoted as the index of the corresponding point and $\left( {Fu_{i}^{T} } \right)_{j}^{2}$ is the square root of the *j*th entity in the $Fu_{i}^{T}$ vector. The 3D fundamental matrix estimation algorithm is shown in Additional file 1: Algorithm S6.

#### Step 4: Reconstruct the 3D matched points locations

In terms of building the localized 3D matrix (density map) using two corresponding images, the 3D locations of the matched points (corresponding) are estimated and calculated. In this step, a typical computer vision algorithm triangulation [53] is used to estimate and calculate the 3D locations of the corresponding points in the 3D space using the estimated 3D matrix from the previous step.

In general, the triangulation algorithm [36] refers to the process that a corresponding point between two images is determining in a 3D space [36]. In another word, triangulation reconstructs the 3D data based on a theory that says each point in an image is corresponding to one single line in a 3D space [52]. In this case, a set of images can be projected in a common 3D point $X$ [52]. The set of lines that are generated by the image points must intersect at the 3D point $X$. The algebra formulation of computing the 3D point $X{ }$ using the triangulation is showing in Eq. (27) [41]:

27 $$X\sim \tau \left( {y_{1}^{^{\prime}} ,y_{2}^{^{\prime}} ,C_{1} ,C_{2} } \right)$$

where $\left[ {y_{1}^{^{\prime}} ,y_{2}^{^{\prime}} } \right]$ are the coordinates of the detected corresponding points in the image, and $[C_{1} ,C_{2} ]$ are the 3D estimated matrix.

The mid-point method [53] is one triangulation method in which each corresponding point in the image $y_{1}^{^{\prime}}$ and $y_{2}^{^{\prime}}$ has one corresponding projected line $L_{1}^{^{\prime}}$ and $L_{2}^{^{\prime}}$ which can be determined by the 3D estimated matrix $[C_{1} ,C_{2} ]$ and computed based on Eq. (28) [53]:

28 $$d\left( {L,X} \right) = Elicedian Distance\left( {L,X} \right)$$

where $d$ is a distance function between the 3D line $L_{1}^{^{\prime}}$ and the 3D point $x$ such that the $X_{est}$ reconstruction point that joins the two projected lines can be calculated using the mid-point method based on the Eq. (29) [53]:

29 $$d\left( {L_{1}^{^{\prime}} ,x} \right)^{2} + d\left( {L_{2}^{^{\prime}} ,x} \right)^{2}$$

The 3D reconstruction of the matched point locations algorithm is shown in Additional file 1: Algorithm S7.

#### Step 5: Metric reconstruction and 3D density map visualization

**Fig. 13:**
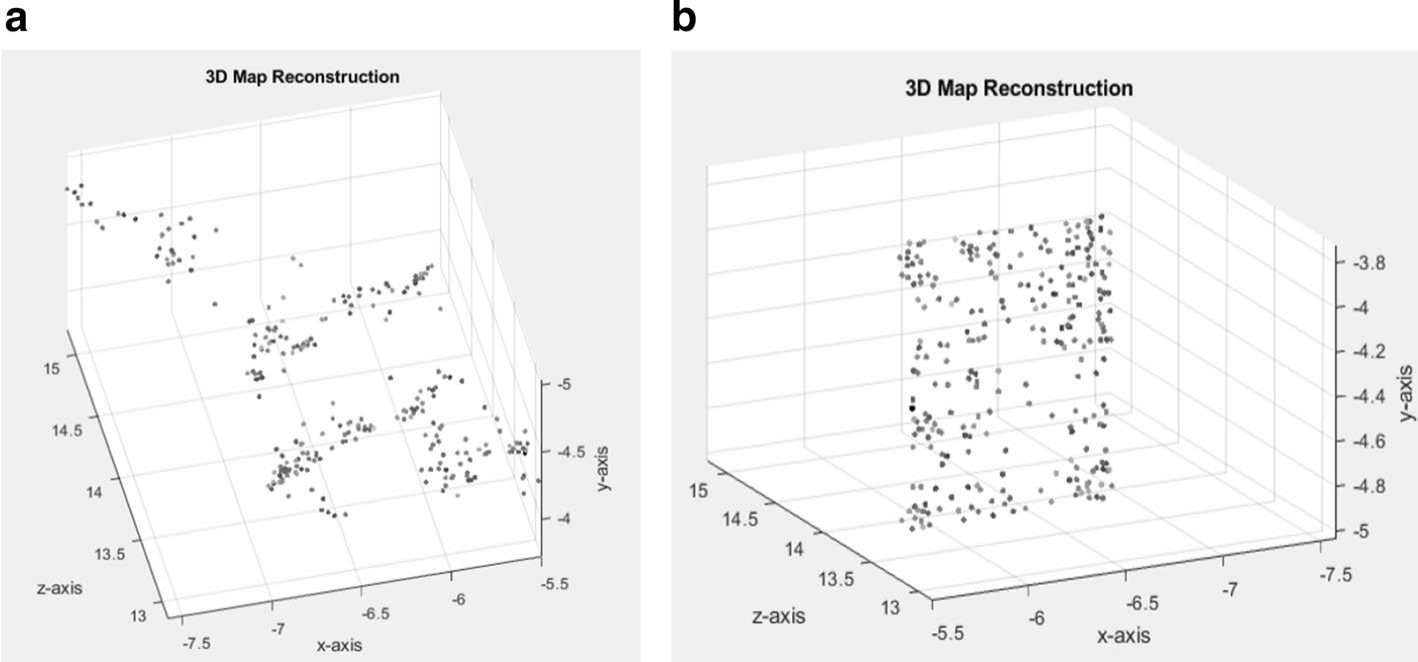
Localized 3D density map reconstruction and visualization. **a** Side view of the localized 3D density map using two side-view particle images only. **b** Frontal view of the localized 3D density map using two side-view particle images only

To visualize the localized 3D density map that is reconstructed based on the first two particle images, we use the MATLAB point cloud visualization function (*pcshow*) and plot the point cloud of the first localized 3D density map of the single side-view protein as is shown in Fig. 13. For instance, Fig. 13a shows the density depth of the first localized 3D density map, while Fig. 13b shows the view of the same localized 3D density map. In terms of computing the second localized 3D density map that is reconstructed between the second and the third particle images, we must reconstruct a new reference particle image as is shown in the main framework of the 3D density map reconstruction (see Figure S3). To reconstruct a new reference image that has important information (corresponding points) between the two images, we use the image fusion approach [54] to gather the important information between the first two particle images, and we need to keep going for the rest of the particles.

In this case, we need to combine the first two particle images to be the reference image. Image fusion is the process to combine two images and inclusion into a new one image [54]. The new image is more accurate and informative than the individual two images since it gathers the corresponding important points (necessary information) between them [54]. The main purpose of doing image fusion is the linear blend [54]. The traditional way (approach) is the linear blend [55]. It combines the two images after converting them to grayscale images and normalized the pixels values in a way that the darkest pixel value is represented by 0 and the lightest one (brightness) is represented by 1 using image Z-score normalization as shown in Eq. (30) [56]:

30 $$x^{\prime} = \frac{{x - \overline{x}}}{\sigma }$$

where $\overline{x}$ is the mean of the intensity pixel values, and $\sigma$ is the standard deviation. Then, the image gradient is computed to detect the directional change in the intensity value of an image as is shown in Eq. (31) [56].

31 $$\nabla f = \left[ {\begin{array}{*{20}c} {g_{x} } \\ {g_{y} } \\ \end{array} } \right] = \left[ {\begin{array}{*{20}c} {\frac{\partial x}{{\partial f}}} \\ {\frac{\partial y}{{\partial f}}} \\ \end{array} } \right]$$

where $\frac{\partial x}{{\partial f}}$ and $\frac{\partial y}{{\partial f}}$ are the gradient in the $x{ }$ and $y$ direction respectively. Then, the regions of the high special variance are combined across one image based on Eq. (32) [56]:

32 $$G = \sqrt {g_{y}^{2} + g_{x}^{2} }$$

**Fig. 14:**
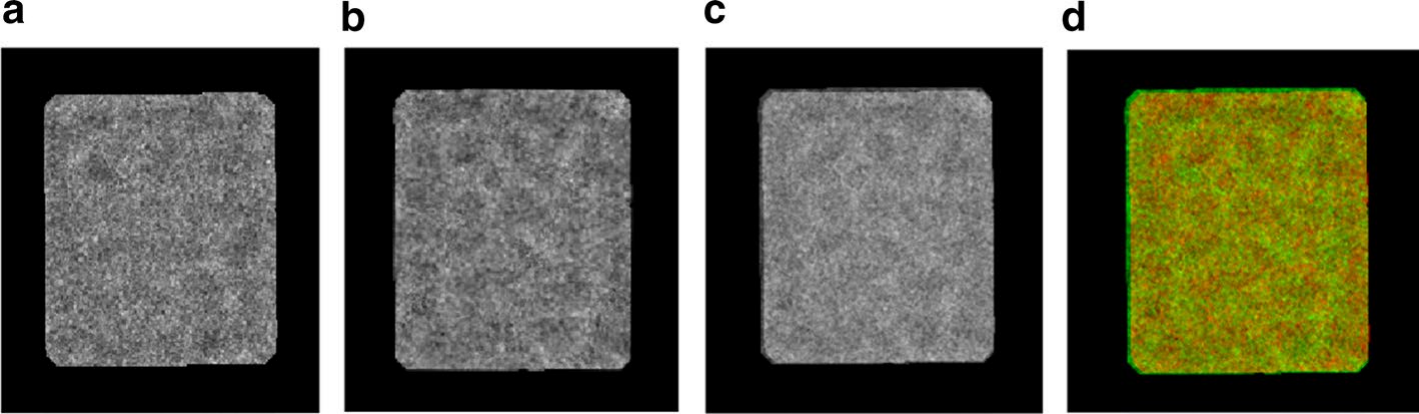
Reference image generation-based image fusion. **a** First original localized aligned particle image. **b** Second localized perfectly aligned particle image. **c** Blended overlay fused particle image, by scaling the intensities of the reference image. **d** Visualized the fused blended (overlay) image using the red channel for the reference particle image, the green channel for the aligned moving image, and yellow channel for the areas of similar intensity between the two images

An important image information based weighted matrix $W$ is calculated. The weighted matrix combines the input gradients $\left| G \right|$ and indicates the desired image output. The basic steps of the image fusion are described in Additional file 1: Algorithm S8. Figure 14 shows an example of particle image reference generation-based image fusion. Figure 14a, b show the first image (reference) and second aligned particle images (moving). Figure 14c shows the blended overlay fused particle image, by scaling the intensities of the reference image (a) and aligned moving image (b) jointly as a single data set. Figure 14d visualized the fused blended (overlay) image using the red channel for the reference particle image, the green channel for the aligned moving image, and the yellow channel for the areas of similar intensity between the two images.

After the new particle reference image generation, the whole process for the second localized 3D density map reconstruction is repeated until the last particle image in the whole dataset is processed. Finally, we average the whole localized 3D density maps to produce the final 3D density map. The 3D reconstruction of the matched point locations algorithm is shown in Additional file 1: Algorithm S9. The whole pipeline of the localized 3D density map reconstruction is illustrated in Additional file 1: Figure S3.

## Results

### Micrograph datasets

Images from two datasets (Apoferritin dataset and Keyhole Limpet Hemocyanin (KLH) dataset) are used to evaluate the method [11]. Two common shapes of protein particles in cryo-EM images are circles and rectangles. Apoferritin dataset [12] uses a multi-frame MRC image format (32 Bit Float). The size of each micrograph is 1240 by 1200 pixels. It consists of 20 micrographs each having 50 frames at 2 electrons/A^2/frame, where the beam energy is 300 kV. The particle shape in this dataset is circular. The Keyhole Limpet Hemocyanin (KLH) dataset from the US National Resource for Automated Molecular Microscopy [12] uses a single frame image format. The size of each micrograph is 2048 by 2048 pixels. It consists of 82 micrographs at 2.2 electrons/A^2/pixel, where the beam energy is 300 120 kV. There are two main types of projection views in this dataset: the top view (circular particle shape) and the side view (square particle shape). The KLH dataset [12] is a standard test dataset for particle picking. The KLH dataset is challenging because of different specimens (different particles) and confounding artifacts (ice contamination, degraded particles, particle aggregates, etc.).

### Experiments of fully automated 2D different particle shapes alignment

The particle picking has been evaluated in our previous work DeepCryoPicker [10]. Here, we focus on evaluating particle alignment and density map reconstruction. The first experimental results of the perfect 2D particle image alignment is based on the perfect 2D mask (square and circular) shapes generation that is shown in Table 1.

**Table 1:** Total number of the particles-selection using fully automated good particles-selection for KLH [12] and Apoferritin [11] datasets

Criteria	Apoferritin top-view	KLH top-view	KLH side-view
Number of micrographs	279	82	
Size of micrograph	1240 × 1200	2048 × 2048	
Resolution (Å)	3.1	9.1	
Total number of picked particles	32,818	2230	1246
Total number of selected particles	24,024	1001	1089
Particle’s patch size	178 × 178	221 × 221	225 × 225

**Fig. 15:**
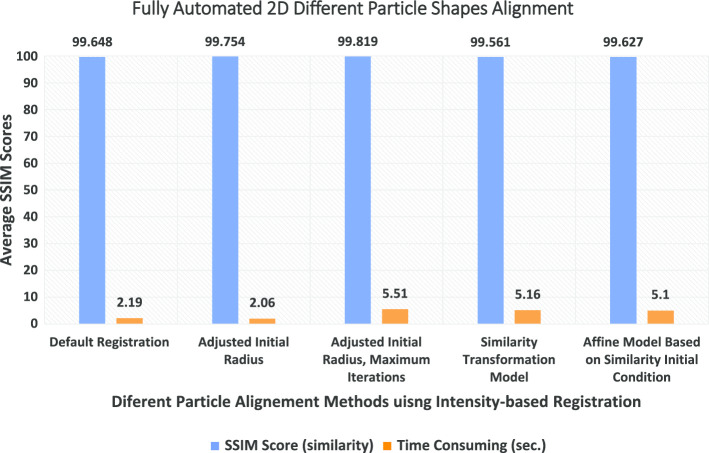
The average similarity scores and the time consuming of different particle alignment methods using the intensity-based image registration approach. The x-axes illustrate the average SSIM score (alignment similarity between each particle and its aligned version), and y-axes illustrate the different particle alignment methods using the intensity-based registration approach

Some experimental results of the perfect 2D particle mask generation (square and circular) KLH [12] and Apoferritin [11] datasets are shown in Additional file 1: Figures S4 and S5. The fully automated side-view particle alignment results using intensity-based registration and perfect generated particle masks are shown in Additional file 1: Figure S6. Also, the experimental results of the regular 2D side-view particles alignment using the KLH dataset [12] are shown in Additional file 1: Figure S6. Also, the experimental results of the localized 2D top and side-view particles alignment using the original and preprocessed particle images from different datasets (KLH [12] and Apoferritin [11]) are shown in Additional file 1: Figure S7, and S8 respectively. The average similarity metric (SSIM) for the fully automated single-particle alignment reaches to 99.819% using the adjusted initial radius image registration with maximum iteration number 300. The corresponding SSIM score for each particle is 100% which is the original view. When we aligned a certain particle, we calculate how much the similarity-based SSIM between the original view and the aligned one. The average SSIM scores for the fully automated single-particle alignment based on different approaches are shown in Table 2. Figure 15 illustrates different fully automated particles alignment methods using intensity-based image registration comparing with the corresponding SSIM scores on the original view. Figure 15 showing the average similarity scores corresponding to the original SSIM scores and the time consuming for each one.

**Table 2:** The average similarity metric scores (SSIM) for the fully automated single-particle alignment

SSIM approach	Similarity	Time consuming
Default registration	99.648	2.19
Adjusted initial radius	99.754	2.06
Adjusted initial radius, maximum iterations	99.819	5.51
Similarity transformation model	99.561	5.16
Affine model based on similarity initial condition	99.627	5.10

Registration method 1 is based on the “default registration model” which registers the two particle images using affine transformation to solve the distortion between the two images includes scaling, rotation. Registration method 2 is based on the “adjusted initial radius model” which improves the particle image registration by adjusting the optimizer and metric configuration properties. Registration method3 is based on the “adjusted initial radius-based maximum iterations model” in which the optimizer controls the maximum number of iterations that the optimizer will be allowed to take. Also allows the registration search to run longer and potentially find better registration results. Registration method 4 is based on the “affine model-based on similarity initial condition model” which registers the particle images by using an “affine” transformation model with the “similarity” results used as an initial condition for the geometric transformation. This model is refined estimate for the registration includes the possibility of shear

### Experiments on fully automated 3D density map reconstruction

**Fig. 16:**
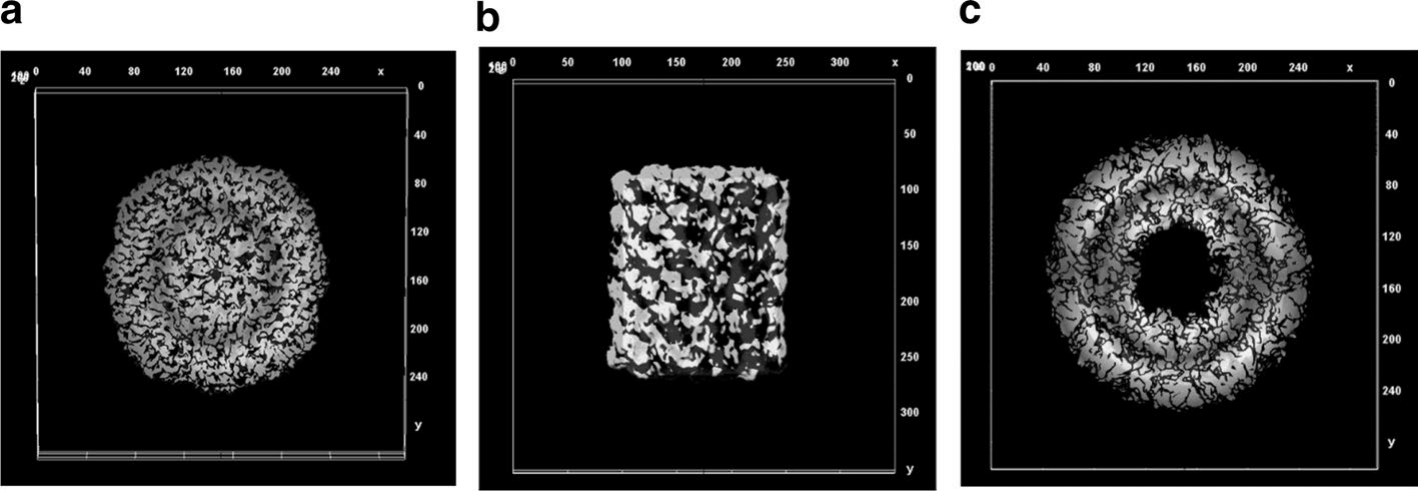
Fully automated 3D density map reconstruction results using the side and top-views particles of the KLH protein molecule and top-view particles of the Apoferritin protein molecule. **a**, **b** Final average side-view 3D density map reconstruction using the preprocessed localized alignment particle images from the KLH dataset respectively [12]. **c** Final average top-view 3D density map reconstruction using the preprocessed localized alignment particle images from the Apoferritin dataset [11]

Two different 3D density maps are reconstructed (top and side-view) [39, 57–59] for two single protein molecules (Apoferritin [11] and KLH [12]). The first 3D density map is automatically reconstructed for the side-view protein molecules using the preprocessed particles from the KLH dataset [12]. The other molecule for the KLH data [12] is the top-view protein molecule. Figure 16a shows the final 3D density map of the Apoferritin [11] top-view protein molecule based on the preprocessed particle images. Figure 16b, c shows the final 3D density map of the KLH [12] top and side-view protein molecule based on using the preprocessed particle images.

## Discussions

**Fig. 17:**
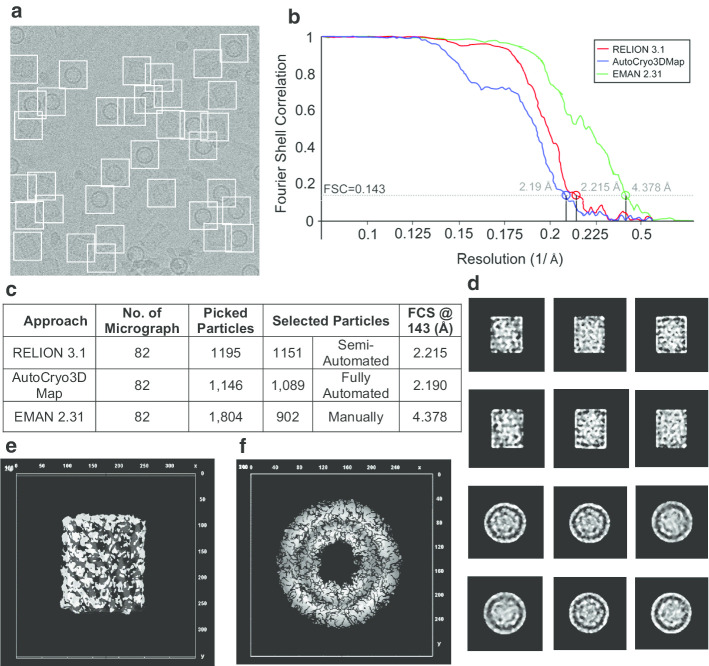
Top and side-view molecular structural analysis using the KLH dataset. **a** Particles picking from a KLH micrograph using DeepCryoPicker [10]. **b** Fourier shell correlation plots for the final 3D reconstruction. The red curve is based on using the RELION 3.1 [8], the blue is based on using Auto3DCryoMap, and the green one is based on using EMAN 2.31 [7]. The average resolution of our 3D density map reconstruction using Auto3DcryoMap is ~ 2.19 Å, whereas that one generated from RELION 3.1 is ~ 2.215 Å and EMAN2 2.31 is ~ 4.378 Å. **c** Summary of particle selection and structural analysis. **d** The preprocessed versions of the original top and side and top-view particles that are used to generate the 3D density map structure. **e**, **f** Top and side views of the KLH 3D density reconstruction obtained with Auto3DCryoMap respectively

We compare the results from the Auto3DCryoMap with two state-of-the-art 3D particle picking and 3D density reconstruction tools—RELION 3.1 [8] and EMAN 2.31 [8]—on different molecular datasets (Apoferritin [11] and KLH [12]). RELION 3.1 [8] initially picks and selects 1195 KLH side-view particles from 82 micrographs and removes 44 particles (see the summary of particle selection and structural analysis table in Fig. 17c. The refinement reconstruction model by RELION 3.1 [8] yields a 3D density map reconstruction at a resolution of ~ 2.215 Å according to the gold-standard FSC = 0.143 criterion [8] (see Fig. 17b). AutoCryo3DMap picks 1,146 KLH side-view particles and selects 1,089 particles (see the summary of particle selection and structural analysis table in Fig. 17c). The preprocessed version of the selected particles is used for the fully automated alignment (see Fig. 17d) yielding a cryo-EM structure of ~ 2.19 Å according to the gold-standard FSC = 0.143 criterion [64] (see Fig. 17b), while EMAN 2.31 [7] produces a 3D density map reconstruction of ~ 4.378 Å according to the gold-standard FSC = 0.143 [8] (see Fig. 17b).

**Fig. 18:**
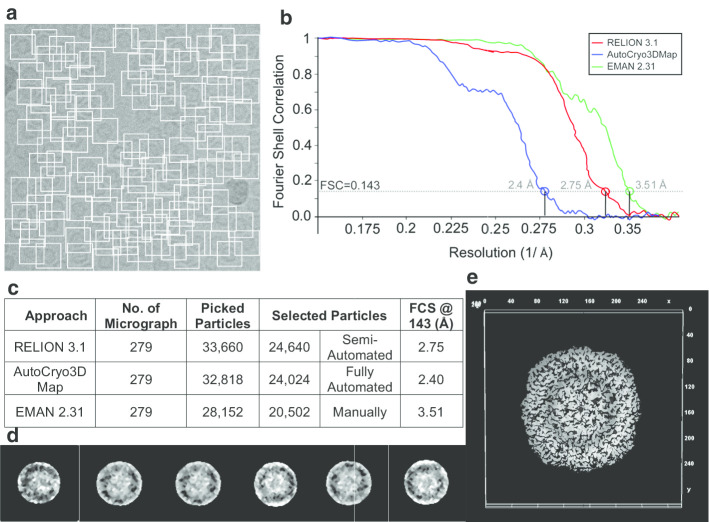
Top-view molecular structural analysis using the Apoferritin dataset. **a** Particles picking from the Apoferritin micrograph using DeepCryoPicker [10]. **b** Fourier shell correlation plots for the final 3D reconstruction. The red curve is based on using the RELION 3.1 [8], the blue is based on using Auto3DCryoMap, and the green one is based on using EMAN 2.31 [7]. The average resolution of our 3D density map reconstruction using Auto3DcryoMap is ~ 2.4 Å, whereas that one generated from RELION is ~ 2.75 Å and EMAN 2.31 is ~ 3.51 Å. **c** Summary of particle selection and structural analysis. **d** The preprocessed versions of the top-view particles that are used to generate the 3D density map structure. **e** 3D density map reconstruction of Apoferritin top-view protein that is obtained by the Auto3DCryoMap

Moreover, for different molecular views, RELION 3.1 [8] picks 33,660 particles and selects 24,640 good Apoferritin top-view particles from 279 micrographs (see the summary of particle selection and structural analysis table in Fig. 18c). The refinement reconstruction model by RELION 3.1 [8] yields a 3D density map reconstruction of Apoferritin at a resolution of 2.75 Å according to the FSC = 0.143 [8] (see Fig. 18b). AutoCryo3DMap automatically selects 32,818 good particles from 24,024 total Apoferritin top-view particles (see the summary of particle selection and structural analysis table in Fig. 18e). AutoCryo3DMap constructs a top-view cryo-EM structure of 2.4 Å, while EMAN 2.31 [7] yields a 3D density map reconstruction at a resolution of 3.51 Å according to the gold-standard FSC = 0.143 [8] (see Fig. 18f).

## Conclusions

We introduce Auto3DCryoMap, a fully automated approach for cryo-EM 3D density maps reconstruction-based deep supervised and unsupervised learning approaches. It uses the fully automated unsupervised learning algorithm ICB [13] to generate 2D particle shapes that are used for the fully perfect particle alignment. Also, the perfect 2D particle images are used to produce localized aligned particle images. We show that the Auto3DCryoMap is able to accurately align top-view and side-view particle shapes. From only a few thousand aligned particle images, Auto3DCryoMap is able to build a decent 3D density map. In contrast, existing tools require hundreds of thousands of particle images. Finally, by using the preprocessed particle images, Auto3DCryoMap reconstructs a better 3D density map than using the original particle images. In the future, we plan to extend our methods to reconstruct 3D density maps of particles with irregular shapes.

## Supplementary information

**Additional file 1.** The supplementary data of Auto3DCryoMap.

## Data Availability

The datasets used in this study and the source code of Auto3DCryoMap are available at https://github.com/jianlin-cheng/DeepCryoMap.

## Abbreviations

Cryo-EM: Cryo-electron microscopy
Micrograph: Digital image taken through a microscope
DeepCryoPicker: Fully automated deep neural network for single protein particle picking in cryo-EM
AutoCryoPicker: An unsupervised learning approach for fully automated single particle picking in cryo-EM image
MRC: Medical Research Council
PNG: Portable network graphic
