# Correction to: Auto3DCryoMap: an automated particle alignment approach for 3D cryo-EM density map reconstruction

**DOI:** 10.1186/s12859-022-04630-0

**Published:** 2022-03-15

**Authors:** Adil Al-Azzawi, Anes Ouadou, Ye Duan, Jianlin Cheng

**Affiliations:** Electrical Engineering and Computer Science Department, University of Missouri, Columbia, MO 65211 USA

## Correction to: BMC Bioinformatics 2020, 21(Suppl 21):534 10.1186/s12859-020-03885-9

Following publication of the original article [1], the authors received valuable feedback from readers in the field regarding our paper that was published in a special issue of BMC Bioinformatics in 2020 (BMC Bioinformatics, 21(S21):534, 2020), we discovered some visualization errors and typos in two Figures (Figs. 17 and 18) in the published manuscript. The Fourier Shell Correlation (FSC) values in Figs. 17b and 18b in the published paper were plotted in the incorrect order by mistake. The error only occurred in visualizing the data, while the original raw data are still the same as before. Therefore, we replot the data to create a new version of Figs. 17 and 18 and fix some typos (e.g., the title of the x-axis of Figs. 17b and 18b) according to the feedback. Figures 17 and 18 are given below.

**Fig. 17:**
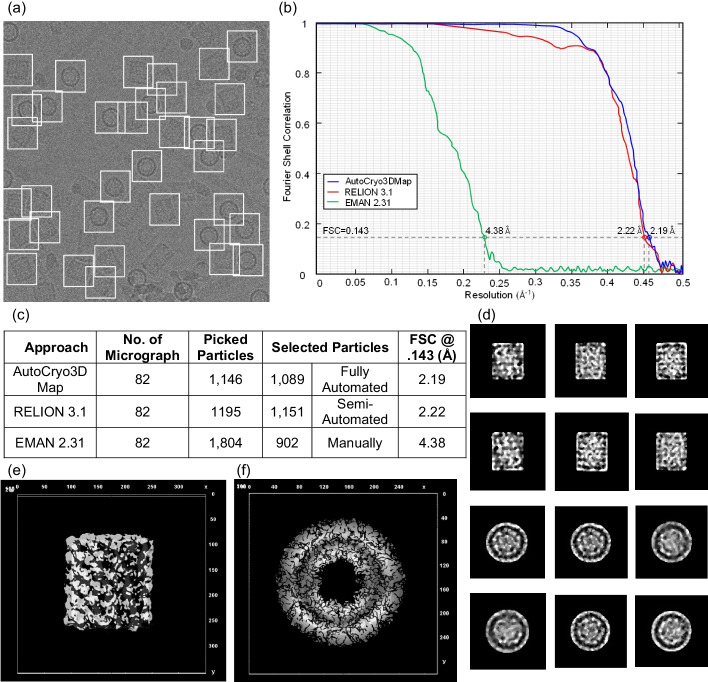
Top and side-view molecular structural analysis using the KLH dataset. **a** Particles picking from a KLH micrograph using DeepCryoPicker [10]. **b** Fourier shell correlation plots for the final 3D reconstruction. The red curve is based on using the RELION 3.1 [8], the blue is based on using Auto3DCryoMap, and the green one is based on using EMAN 2.31 [7]. The average resolution of our 3D density map reconstruction using Auto3DcryoMap is ~ 2.19 Å, whereas that one generated from RELION 3.1 is ~ 2.215 Å and EMAN2 2.31 is ~ 4.378 Å. **c** Summary of particle selection and structural analysis. **d** The preprocessed versions of the original top and side and top-view particles that are used to generate the 3D density map structure. **e**, **f** Top and side views of the KLH 3D density reconstruction obtained with Auto3DCryoMap respectively

**Fig. 18:**
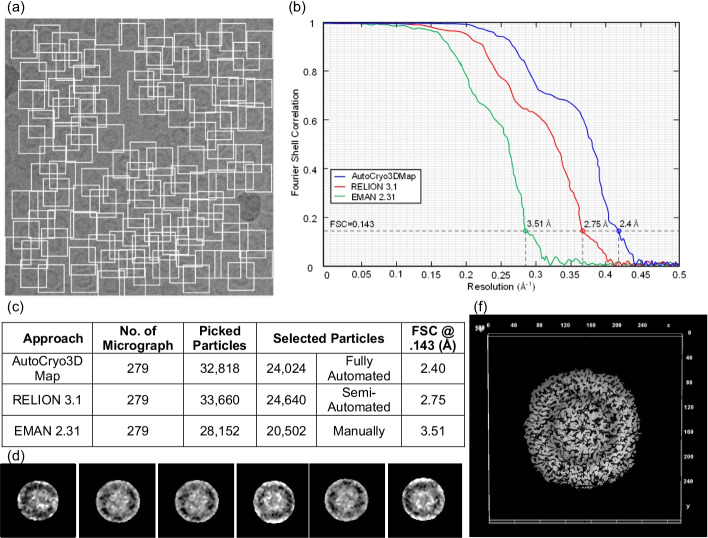
Top-view molecular structural analysis using the Apoferritin dataset. **a** Particles picking from the Apoferritin micrograph using DeepCryoPicker [10]. **b** Fourier shell correlation plots for the final 3D reconstruction. The red curve is based on using the RELION 3.1 [8], the blue is based on using Auto3DCryoMap, and the green one is based on using EMAN 2.31 [7]. The average resolution of our 3D density map reconstruction using Auto3DcryoMap is ~ 2.4 Å, whereas that one generated from RELION is ~ 2.75 Å and EMAN 2.31 is ~ 3.51 Å. **c** Summary of particle selection and structural analysis. **d** The preprocessed versions of the top-view particles that are used to generate the 3D density map structure. **e** 3D density map reconstruction of Apoferritin top-view protein that is obtained by the Auto3DCryoMap
