# Supplementary material for: Auto3DCryoMap: an automated particle alignment approach for 3D cryo-EM density map reconstruction
Source: BMC Bioinformatics. 2020 Dec 28;21(Suppl 21):534. doi: 10.1186/s12859-020-03885-9 (PMC7768659; doi:10.1186/s12859-020-03885-9)
Supplement: Supplementary file 1 — Additional file 1. The supplementary data of Auto3DCryoMap. [file 12859_2020_3885_MOESM1_ESM.docx]

## Additional file 1: Algorithms:

| **Algorithm S1** Fully Automated Perfect 2D Side-View Particles Selection |
| --- |
| 1. Label each connected components (objects) in the cleaned image and extract the total number of objects including a list of pixel locations for each one using MATLAB function (*bwlabel*). |
| 1. **for** each object in the cleaned clustered image **do** |
| 1. Smooth each object shape specific kernel size=5x5. |
| 1. **end for** |
| 1. Determine the connected components (objects) in the image, including a list of pixel locations for each one using MATLAB function (*bwlabel).* |
| 1. Measure set of properties specified by properties for each 8-connected component in the binary image using MATLAB function (*regionprops).* |
| 1. Calculate the ferret properties |
| 1. **for** each object in the cleaned clustered image **do** |
| 1. $P_{\mathrm{list}}\leftarrow PixelList(objects)$ **/***Convert each object pixel to coordinates as an x-y order including a list of pixel locations for each one using MATLAB function (*PixelList*)*/   |
| 1. $P_{\mathrm{hull}}\leftarrow PixelHull(P_{\mathrm{list}})$ /*Extract the pixel hull diamond shapes using MATLAB function (*PixelHull*) */   |
| 1. $P_{\mathrm{pairs}}\leftarrow VerticPair(P_{\mathrm{hull}})$ **/***Determine the maximum Feret diameter and its orientation (maximum diameter) */   |
| 1. $\mathrm{Feter}_{\mathrm{dimeter}}\leftarrow Min(P_{\mathrm{pairs}})$ /*Computes the minimum ferret diameter*/   |
| 1. $\mathrm{Area}_{\mathrm{bounding}}\leftarrow Min\left( \mathrm{Feter}_{\dim} \right)$/*Extract the minimum bounding box area*/   |
| 1. **end for** |
| 1. **for** each object in the cleaned clustered image **do** |
| 1. Determine the connected components (objects) in the image, including a list of pixel locations for each one and extract the centroid is the horizontal coordinate (or x-coordinate) and vertical coordinate (or y-coordinate) using MATLAB function (*regionprops('centroid’*)). |
| 1. Extract the bounding box dimension for each object (perfect square). |
| 1. Determine and extract the 2D convex hull of the points $(X,Y)$ for each object (particle) /*$X$ and $Y$ are column-vectors which presents a vector of point indices arranged in a counter-clockwise cycle around the hull */ |
| 1. **end for** |
| 1. Construct the final cleaned output image containing only the perfect square objects classes occurring in each image by inserting a perfect square shape using the same dimension points$(X,Y)$ and MATLAb function (*Insert*) |
| 1. Determine and measure the connected components properties of all objects in the cleaned binary image including a list of pixel locations for each one using MATLAB function (*bwlabel).* |
| 1. Determine and eliminate the outliers object using MATLAb function (*outliers*). |
| 1. **for** each object in the cleaned clustered image **do** |
| 1. Determine the average region area of each connected component (object) using MATLAB function (*regionprops('Area')*). |
| 1. **end for** |
| 1. **for** each object in the cleaned clustered image **do** |
| 1. Determine the region area of each connected component (object) using MATLAB function (*regionprops('Area')*). |
| 1. keep objects that are less that the average rectangle region area. |
| 1. Extract each object position using MATLAB function (*ismember*). |
| 1. **end for** |
| 1. Determine the connected components (objects) in the image, including a list of pixel locations for each one and extract the centroid is the horizontal coordinate (or x-coordinate) and vertical coordinate (or y-coordinate) using MATLAB function (*regionprops('centroid’*)). |
| 1. Draw all bounding box for each discontinuous region (rectangle region area). |
| 1. Construct the cleaned output image containing only the perfect square object classes occurring in each image. |

| **Algorithm S2** Fully Automated 2D Particle Binary Mask Post-processing |
| --- |
| 1. **input:** $I_{c}$ /*cluster cryo-EM image */ |
| 1. **return:** $I_{cc}$ /*cleaned cluster image */ |
| 1. $\boldsymbol{I}_{\boldsymbol{c}\mathbf{1}}\leftarrow imopen(I_{c})$ /* Generate an intermediate clustered image by enlarge the small object using the image opening according to Equation (11) */. |
| 1. $\boldsymbol{L}\leftarrow bwlabel(I_{c1})$ /* Label each object in the cluster image using MATLAB function ($bwlabel$) */. |
| 1. **for** i=1 to $L$ **do** /* for each object in the intermediate clustered image*/ |
| 1. $\mathbf{I}_{\mathbf{object}}\leftarrow state(L(k))$ /* determine the connected components (objects) in the image, including a list of indexing pixel locations for each one using MATLAB function (regionprops) */. |
| 1. $\mathbf{I}_{\mathbf{object}}\leftarrow bwareaopen\left( state(L(k)) \right)$ /*remove the object that has not a fully connected edge using MATLAB function (bwareaopen)*/. |
| 1. **end for** |
| 1. $\mathrm{obj}_{\mathrm{number}}\leftarrow is member(I_{\mathrm{object}})$ /*extract the number of object (particles)*/ |
| 1. $L\leftarrow bwlabel$ /*label each object (particle)*/ |
| 1. **for** $i$=1 to $L$ **do** /* for each object (particles) */ |
| 1. Do size filtering and roundness filtering |
| 1. $Areas \leftarrow[props.Area]$ /* Determine the region area of each connected component (object) using MATLAB function (region props('Area')) */ |
| 1. $\mathrm{Threshol}d_{\mathrm{area}}\leftarrow50000$ /*determine the value of the average object. */ |
| 1. $keeperObjects\leftarrow threshold_{\mathrm{area}}$ /* Keep objects that less than or equal to the average object’s using MATLAB function (bwareaopen) */. |
| 1. Get actual index numbers instead of a logical vector |
| 1. $I_{c2}\leftarrow$produce new binary image with only the small, round objects in it |
| 1. $I_{\mathrm{cc}}\leftarrow bwareaopen(I_{c})$ /*remove the object that has not a fully connected edge*/ |
| 1. **end for** |
| 1. Construct the output image containing only the object circular “roundness” object classes in each image. |

| **Algorithm S3** Fully Automated Perfect “good” 2D Top-view Particle Selection |
| --- |
| 1. Extract the convex hull points of the outer circular object ring points |
| 1. Remove the outer circular object ring points-based using morphological image operation “*imerode*” with structural size=10. |
| 1. Multiply the erosion image with the original binary mask |
| 1. Extract the convex hull of the inner circular object ring points |
| 1. Extract the area between the inner and the outer convex hull of the circular object |
| 1. Generate a gaussian kernel (mask) using $G_{\sigma}=\frac{1}{\sqrt{2{\pi\sigma}^{2}}}e^{\left( -\frac{m^{2}+n^{2}}{2\sigma^{2}} \right)}$, where $\sigma$ is$\sigma$ is the sigma (which represents the signal width) , $m$, and $n$ is the image dimension. |
| 1. Smooth the input image with a Gaussian filter to reduce noise and unwanted details and textures by using $g\left( m,n \right)=G_{\sigma}(m,n)\times f\left( m,n \right)$ where $g\left( m,n \right)$ is the output image, $f(m,n)$ is the original input image, and $G_{\sigma}$is the gaussian kernel (mask). |
| 1. Compute gradient of $g(m,n)$ using any of the gradient operators to get $M$ by using $M(m,n)=\sqrt{(g_{m}^{2} (m,n)+) g_{n}^{2} (m,n)}$ 2. Where $g_{m}$ is the gradient in the x-axis direction, $g_{n}$ is the gradient in the y-axis direction. |
| 1. Threshold the gradient $M$ by $M_{T}\left( m,n \right)=\left\{ \begin{aligned} M\left( m,n \right) if M\left( m,n \right)>T \\ 0 Otherwise \end{aligned} \right.$ |
| 1. /* *Hough Transform Begin**/ |
| 1. **for** each edge point **do** |
| 1. Draw a circle with centre $(x,y)$ in the edge point with $r$ where $(x,y)$ is the image pixels with position $x,$ and $y$, $r$ is the circular radius. |
| 1. Increment all coordinates $(x,y)$ that the perimeter of the circle passes through in the accumulator. |
| 1. Find one or several maxima in the accumulator |
| 1. Map the found parameters $(r,a,b)$corresponding to the maxima back to the original image, where $a$, and $b$ is the centre of the maxima. |
| 1. **end for** |
| 1. /* *Hough Transform End**/ |
| 1. Use the detected centre to construct a perfect circular object with the average dimeter. |
| 1. $\boldsymbol{L}\leftarrow bwlabel(I_{c1})$ /* Label each object in the cluster image using MATLAB function ($bwlabel$) */. |
| 1. **for** i=1 to $L$ **do** /* for each object in the intermediate clustered image*/ |
| 1. $\mathbf{I}_{\mathbf{object}}\leftarrow state(L(k))$ /* determine the connected components (objects) in the image, including a list of indexing pixel locations for each one using MATLAB function (regionprops) */. |
| 1. $\mathbf{I}_{\mathbf{object}}\leftarrow bwareaopen\left( state(L(k)) \right)$ /*remove the object that has not a fully connected edge using MATLAB function (bwareaopen)*/. |
| 1. **end for** |
| 1. $\mathrm{obj}_{\mathrm{number}}\leftarrow is member(I_{\mathrm{object}})$ /*extract the number of object (particles)*/ |
| 1. $L\leftarrow bwlabel$ /*label each object (particle)*/ |
| 1. **for** $i$=1 to $L$ **do** /* for each object (particles) */ |
| 1. Do size filtering and roundness filtering |
| 1. $Areas \leftarrow[props.Area]$ /* Determine the region area of each connected component (object) using MATLAB function (region props('Area')) */ |
| 1. $Perimeters \leftarrow[props.Perimeter]$ /* Determine the region perimeters of each connected component (object) using MATLAB function (region props (Perimeter)) */ |
| 1. $Circularities \leftarrow allPerimeters^2/((4\times pi\times allAreas))$ /* Determine the region circularitiesof each connected component (object) using Equation (12). |
| 1. $\mathrm{Threshol}d_{\mathrm{area}}\leftarrow50000$ /*determine the average objects "roundness" circularities value. */ |
| 1. $keeperObjects\leftarrow circularities < 3 \& Areas < threshold_{\mathrm{area}}$ /* Keep objects that less than or equal to the average object’s "roundness" circularities value using MATLAB function (bwareaopen) */. |
| 1. Get actual index numbers instead of a logical vector |
| 1. $I_{c2}\leftarrow$produce new binary image with only the small, round objects in it |
| 1. $I_{\mathrm{cc}}\leftarrow bwareaopen(I_{c})$ /*remove the object that has not a fully connected edge*/ |
| 1. **end for** |
| 1. Construct the output image containing the perfect circular “roundness” object |

| **Algorithm S4** Fully Automated 2D Side-View Particle Alignment |
| --- |
| 1. /**Reference mask image reconstruction**/ |
| 1. Import the whole binary mask particle images |
| 1. **for** each particle image **do** /* for each binary mask image (particles) */ |
| 1. /* *Hough Transform Begin**/ |
| 1. **for** each edge point **do** |
| 1. Draw a circle with centre $(x,y)$ in the edge point with $r$ where $(x,y)$ is the image pixels with position $x,$ and $y$, $r$ is the circular radius. |
| 1. Increment all coordinates $(x,y)$ that the perimeter of the circle passes through in the accumulator. |
| 1. Find one or several maxima in the accumulator |
| 1. Map the found parameters $(r,a,b)$corresponding to the maxima back to the original image, where $a$, and $b$ is the centre of the maxima. |
| 1. **end for** |
| 1. /* *Hough Transform End**/ |
| 1. Compute the similarity metric using Equation (9) |
| 1. Specify the gematrical transformation. |
| 1. Do the [intensity-based image registration](https://www.mathworks.com/help/images/create-an-optimizer-and-metric-for-intensity-based-image-registration.html) using Equations (10)-(20). |
| 1. /* *Particle Binary Mask Based Image Alignment Begin**/ |
| 1. **Repeat** |
| 1. Align the moving binary mask particle image $I_{binary}\left( x,y \right)$ using Equation (6) |
| 1. $I_{binary\_aligned}\left( x,y \right)=I_{binary\_moved}(T_{opt}=\underset{T\in\mathcal{T}}{\mathrm{argmax}} S\left( I_{binary\_Refrence},I_{Moved\_orginal}\left( T \right) \right))$ |
| 1. **until** convergence or reach the maximum iterations number |
| 1. /* *Particle Binary Mask Based Image Alignment End**/ |
| 1. Extract the angle$\theta_{aligned}$ between the x-axis and the major axis of the object that has the same second-moments as the region on the aligned binary image, returned as a scalar. |
| 1. Extract the orientation $\theta_{orginal}$ angle between the horizontal dotted line and the major axis in the original binary mask |
| 1. $\theta_{orientaion}=\left\vert\theta_{aligned}-\theta_{orginal} \right\vert$ |
| 1. $I_{orginal\_aligned}\left( x,y \right)=I_{original\_moved}(R_{\theta_{orientaion}}(x,y) )$ /* use the extracted angle $\theta_{orientaion}$ to rotate the original particle image |
| 1. **end** |
| 1. $I_{localized}=I_{orginal}\times I_{mask}$/*Construct the localized original aligned image $I_{aligned}$ by multiply the aligned particle image by the aligned particle mask*/ |

| **Algorithm S5** Fully Automated 2D Top-View Particle Alignment |
| --- |
| 1. Import the binary mask particle images $I_{mask}$ |
| 1. Import the original particle images $I_{orginal}$ |
| 1. $I_{localized}=I_{orginal}\times I_{mask}$/*Construct the localized original aligned image $I_{aligned}$ by multiply the aligned particle image by the aligned particle mask*/ |
| 1. **for** each particle image **do** /* for each binary mask image (particles) */ |
| 1. /* *Hough Transform Begin**/ |
| 1. **for** each edge point **do** |
| 1. Draw a circle with centre $(x,y)$ in the edge point with $r$ where $(x,y)$ is the image pixels with position $x,$ and $y$, $r$ is the circular radius. |
| 1. Increment all coordinates $(x,y)$ that the perimeter of the circle passes through in the accumulator. |
| 1. Find one or several maxima in the accumulator |
| 1. Map the found parameters $(r,a,b)$corresponding to the maxima back to the original image, where $a$, and $b$ is the centre of the maxima. |
| 1. **end for** |
| 1. /* *Hough Transform End**/ |
| 1. $\left[ x,y \right]\leftarrow Hough Transform\left( BinaryMask \right)$ /*Extract the centre of the binary circular binary object (particle mask) */.   |
| 1. $\left[ x \right]\leftarrow x+factor$, $\left[ y \right]\leftarrow y+factor$ /* increase the dimensions of the candidate box using the same factor value */ |
| 1. Draw all bounding box for each discontinuous region (rectangle region area). |

| **Algorithm S6** 3D Fundamental Matrix Estimation |
| --- |
| 1. Initialize the 3D fundamental matrix $F$, 3-by-3 matrix of zeros. |
| 1. Import List1 and List /*corresponding points list in the first and second particle image*/ |
| 1. Set the loop counter $n$, to zero, and the number of loops $N$, to the number of random trials specified |
| 1. **while** $i\leq$ $N$ **do** /* Loop through the following steps */ |
| 1. **for** each edge point **do** |
| 1. Randomly select 8 pairs of points from List1 and List2. |
| 1. Use the selected 8 points to compute a fundamental matrix, $f$, by using the normalized 8-point algorithm. |
| 1. Compute the fitness of $f$ for all points in List1 and List2 using Equation (22) and (23). |
| 1. **If** fitness of $f$ is better than $F$ |
| 1. $F\leftarrow f.$ |
| 1. **end if** |
| 1. update $N$ using Equation (24) |
| 1. **end while** |
| 1. $n=n+1$ |

| **Algorithm S7** Reconstruct the 3D Matched Points Locations |
| --- |
| 1. $I_{R}\leftarrow I[i]$ /*Import the first particle image from the whole dataset and Let assume the first particle image is the reference image $I_{R}$ */ |
| 1. $I_{M}\leftarrow I[i]$ /*Import the second particle image from the whole dataset and assume the next particle image is the moving image */ |
| 1. $I_{M}\leftarrow I[i]$ /*Assume the next particle image is the moving image*/ |
| 1. ${List}_{1}\leftarrow Detect_{point}\left( I_{R} \right)$ /* Detect a sparse set of points (detects corners) in the reference image*/ |
| 1. ${List}_{2}\leftarrow Detect_{point}\left( I_{M} \right)$ /* Detect a sparse set of points (detects corners) in the moving image*/ |
| 1. ${Track}_{points}\leftarrow KLTt_{point}\left( {List}_{1},{List}_{2} \right)$ /* Match and track a sparse set of points between the two images using (KLT) tracking points algorithm*/ |
| 1. $F[C_{1},C_{2}]\leftarrow{3D}_{Fundematal Matrix}\left( {List}_{1},{List}_{2} \right)$ /*Estimate the fundamental matrix*/ |
| 1. ${Match}_{points}[y_{1}^{'},y_{2}^{'}]\leftarrow Detect_{point}\left( I_{R},I_{M} \right)$ /*Match a dense set of points (Re-detect the corners) between the two images*/ |
| 1. $X\sim\tau\left( y_{1}^{'},y_{2}^{'},C_{1},C_{2} \right)$ /*Determine the 3D locations of the matched points using triangulate*/ |
| 1. Recover the actual scale, resulting in a 3D metric reconstruction |

| **Algorithm S8** Fully Automated Particle Image Reference Generation Based Image Fusion |
| --- |
| 1. $I_{1}\leftarrow I_{i}$ /*Import the first particle image from the whole dataset*/ |
| 1. $I_{2}\leftarrow I_{i}$ /*Import the second particle image from the whole dataset*/ |
| 1. $G_{i}\leftarrow{\nabla f(I}_{i})$ /*Find gradient field of the two images*/ |
| 1. $W_{i}\leftarrow\left\vert G_{i} \right\vert$ /*Compute importance image $W_{i}$ from $\left\vert G_{i} \right\vert$*/ |
| 1. **for** each pixel $\left( x,y \right)$ **do** |
| 1. $G(x,y)\leftarrow\sum_{i} \frac{W_{i}(x,y)G_{i}(x,y)}{\sum_{i} W_{i}(x,y)}$ /* Compute mixed gradient field*/ |
| 1. **end for** |
| 1. $I^{'}\leftarrow\left\vert G_{i} \right\vert$ /*Reconstruct image $I$ from gradient field $G$ */ |
| 1. $I^{'}\leftarrow\sum_{i} W_{i}I_{i}$ /*Normalize pixel intensities in $I$ to closely matched*/ |

| **Algorithm S9** Fully Automated Localized 3D Density Map Reconstruction |
| --- |
| 1. $I_{R}\leftarrow I[i]$ /*Import the first particle image from the whole dataset*/ |
| 1. Let assume the first particle image is the reference image $I_{R}$ |
| 1. **for** $i$=2 to the total number of the particle images $N$ **do** |
| 1. $I_{M}\leftarrow I[i]$ /*Assume the next particle image is the moving image*/ |
| 1. ${List}_{1}\leftarrow Detect_{point}\left( I_{R} \right)$ /* Detect a sparse set of points (detects corners) in the reference image*/ |
| 1. ${List}_{2}\leftarrow Detect_{point}\left( I_{M} \right)$ /* Detect a sparse set of points (detects corners) in the moving image*/ |
| 1. ${Track}_{points}\leftarrow KLTt_{point}\left( {List}_{1},{List}_{2} \right)$ /* Match and track a sparse set of points between the two images using (KLT) tracking points algorithm*/ |
| 1. $F[C_{1},C_{2}]\leftarrow{3D}_{Fundematal Matrix}\left( {List}_{1},{List}_{2} \right)$ /*Estimate the fundamental matrix*/ |
| 1. ${Match}_{points}[y_{1}^{'},y_{2}^{'}]\leftarrow Detect_{point}\left( I_{R},I_{M} \right)$ /*Match a dense set of points (Re-detect the corners) between the two images*/ |
| 1. $X_{i}\sim\tau\left( y_{1}^{'},y_{2}^{'},C_{1},C_{2} \right)$ /*Determine the 3D locations of the matched points using triangulate*/ |
| 1. Recover the actual scale, resulting in a 3D metric reconstruction |
| 1. $I_{new\_refrence}\leftarrow{Fusion(I_{R},I}_{M})$ /*produce new reference image by fused the reference and moving image in one image*/ |
| 1. $I_{R}\leftarrow I_{new\_refrence}$ /*Assign the new fused reference image by the old refence image*/ |
| 1. **end for** |
| 1. $X_{final}=\sum_{i=1}^{N} \frac{X_{i}}{N}$ /*Reconstruct the final 3D density map by average the whole localized 3D density maps*/ |

## Additional file 1: Figures:


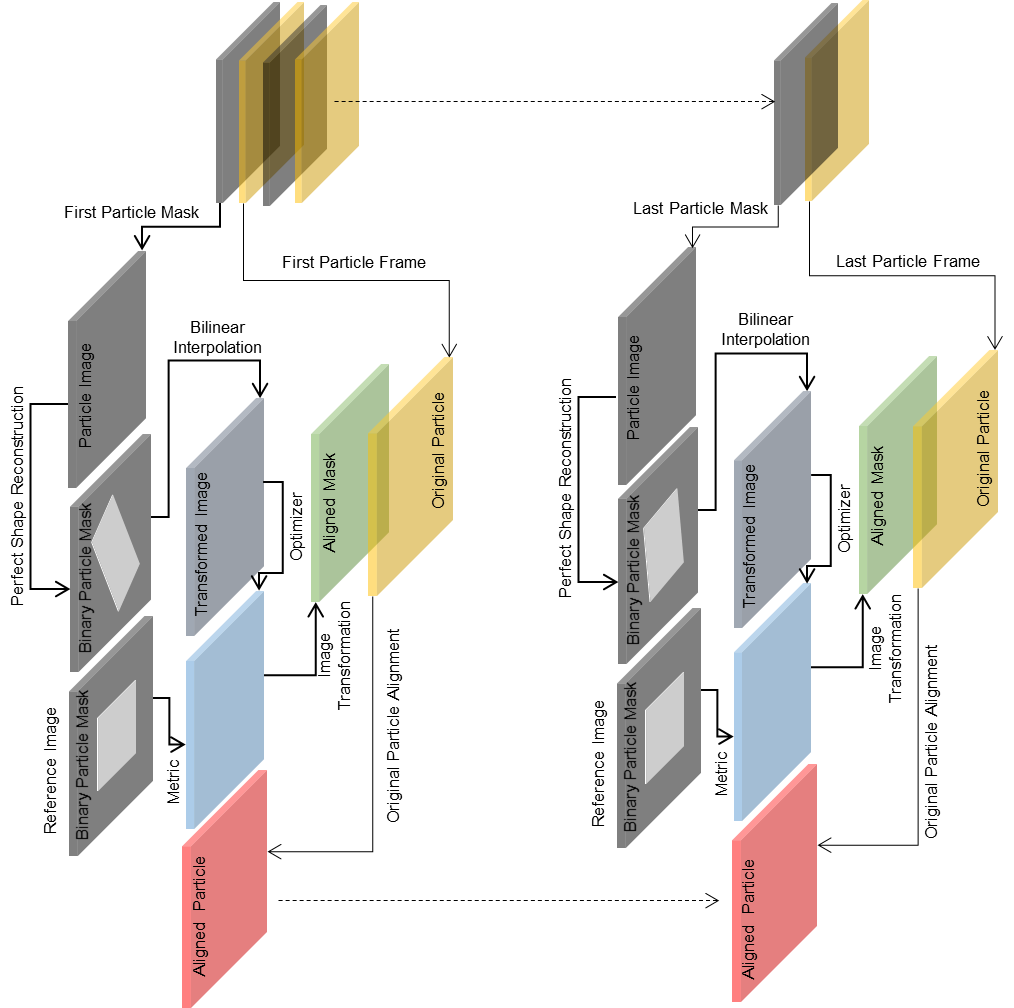


**Figure S1** Fully automated side-view particle alignment framework using intensity-based image registration.


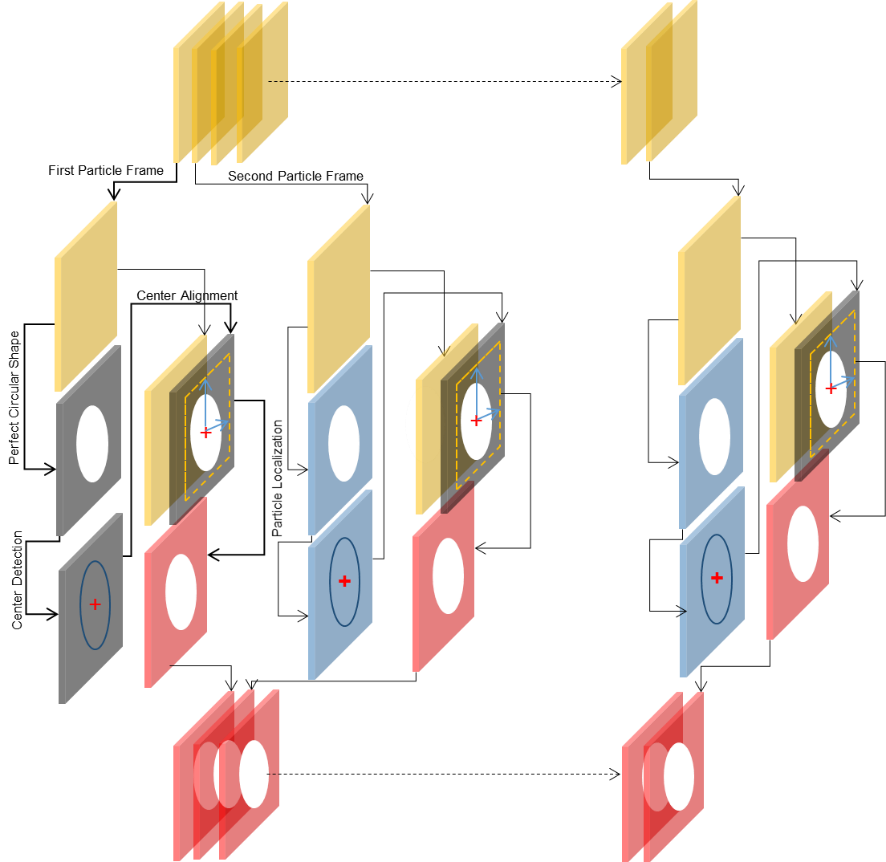


**Figure S2** Fully automated localized 2D top-view particle alignment framework using centralized image alignment based perfect binary 2D image.


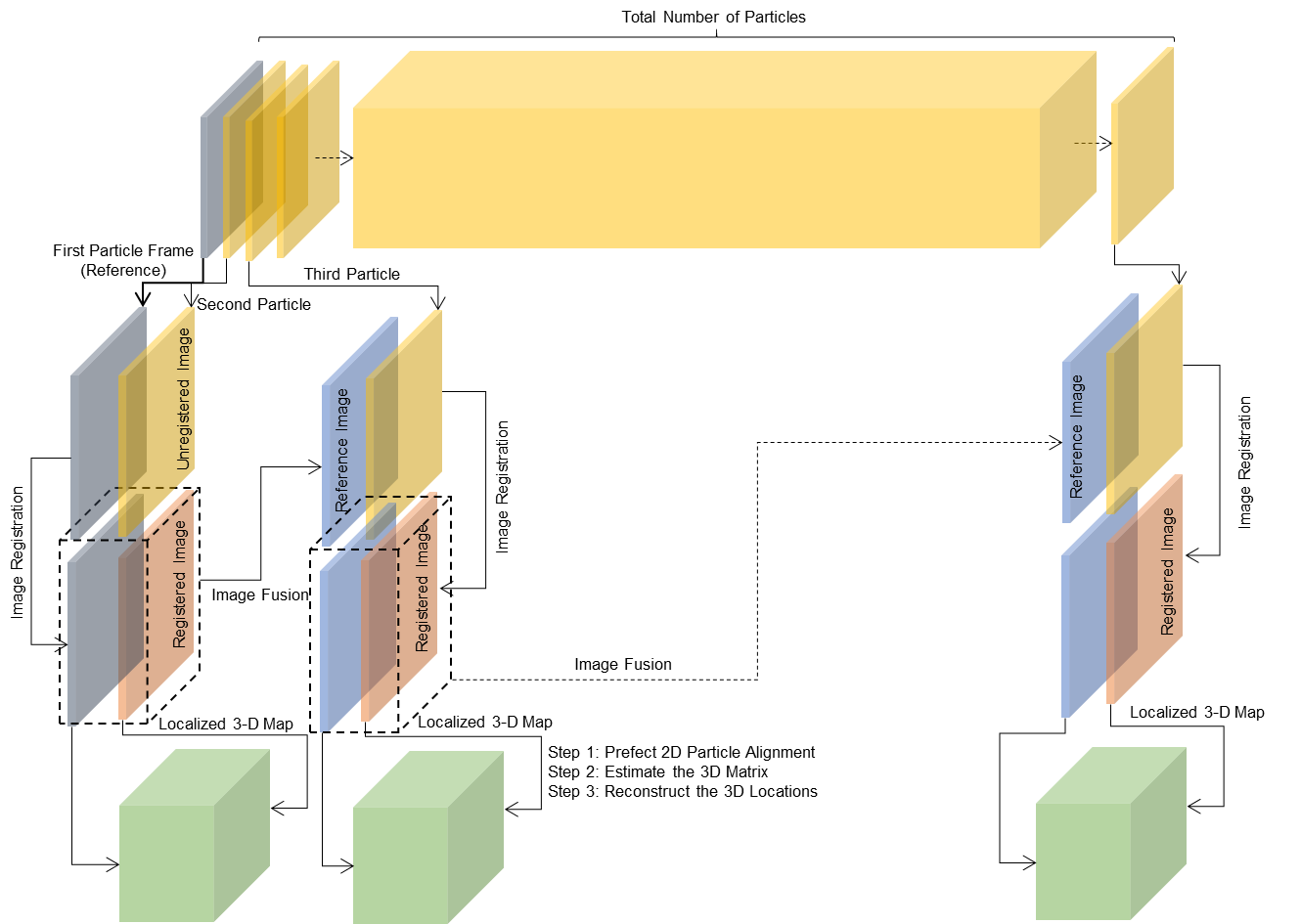


**Figure S3** Localized 3D density map reconstruction framework using structural based motion information.

| 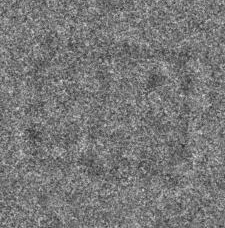 | 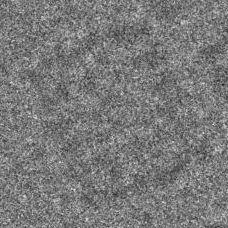 | 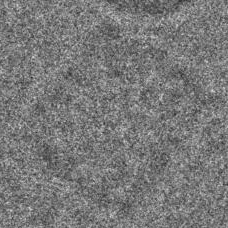 | 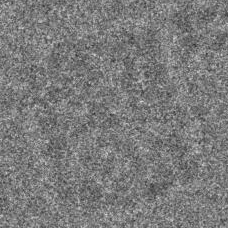 |
| --- | --- | --- | --- |
| (a) | (b) | (c) | (d) |
| 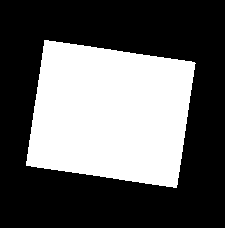 | 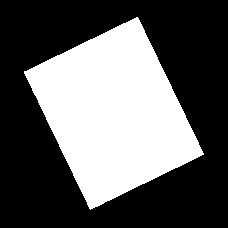 | 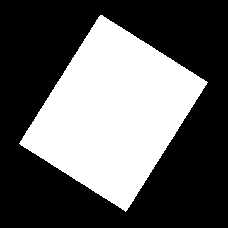 | 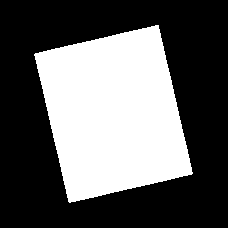 |
| (e) | (f) | (g) | (h) |

**Figure S4** Perfect binary mask generation results for 2d side-view particles. (a)-(d) are the original side-view particle image from KLH dataset [22], (e)-(h) are the perfect binary mask generation for the good 2D particle sample selection.

| 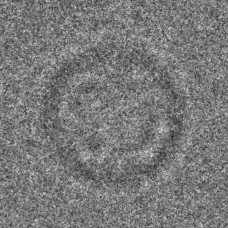 | 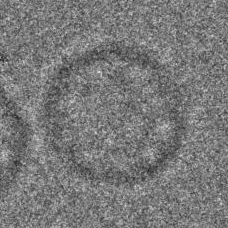 | 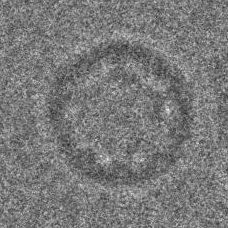 | 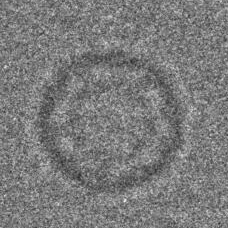 |
| --- | --- | --- | --- |
| (a) | (b) | (c) | (d) |
| 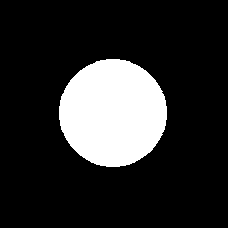 | 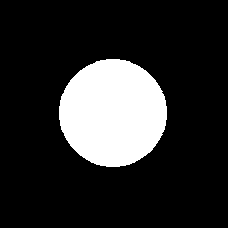 | 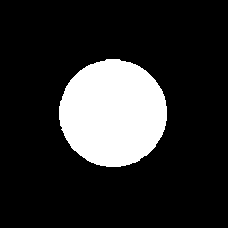 | 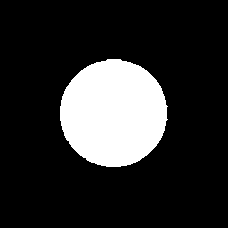 |
| (e) | (f) | (g) | (h) |
| 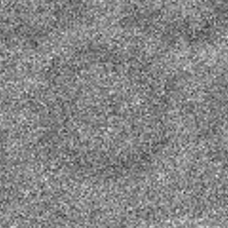 | 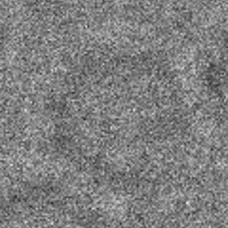 | 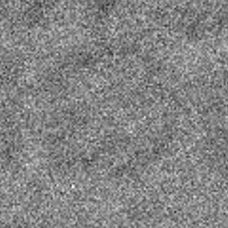 | 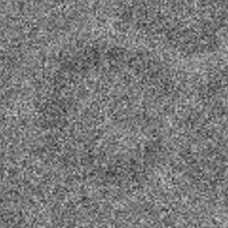 |
| (i) | (j) | (k) | (l) |
| 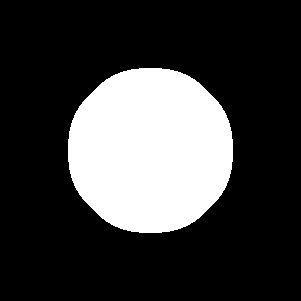 | 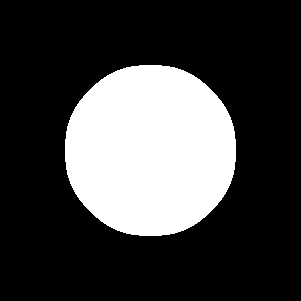 | 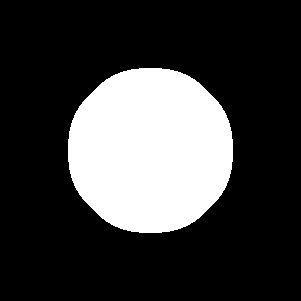 | 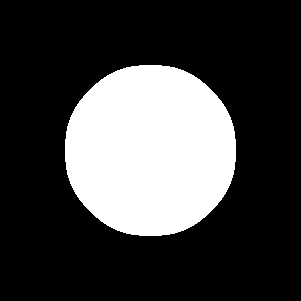 |
| (m) | (n) | (o) | (p) |

**Figure S5** Perfect binary mask generation results for 2d top-view particles. (a)-(d) the original side-view particle image from KLH dataset [22], (i)-(l) the original side-view particle image from Apoferritin dataset [21], (e)-(h) and (m)-(p) the perfect binary mask generation for the good 2D particle sample selection.

| 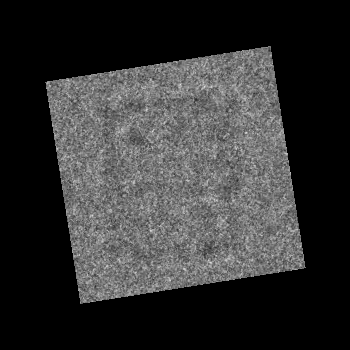 | 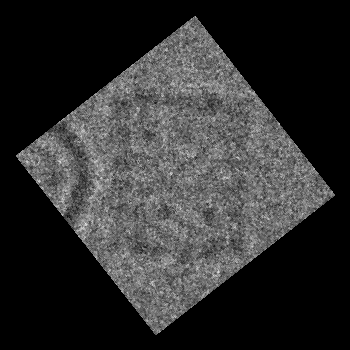 | 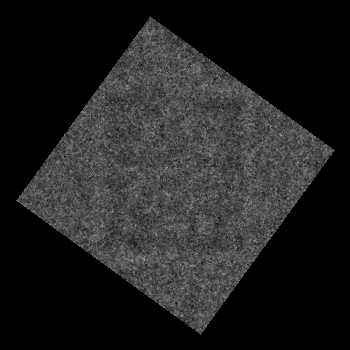 | 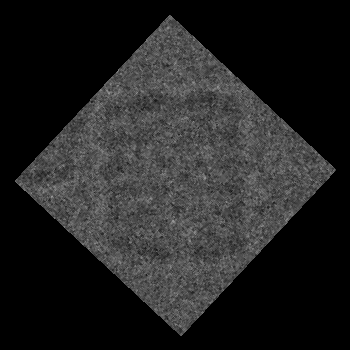 |
| --- | --- | --- | --- |
| 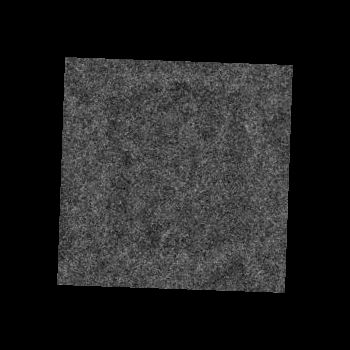 | 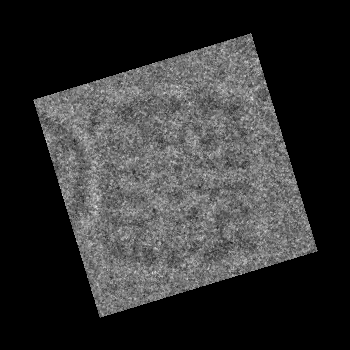 | 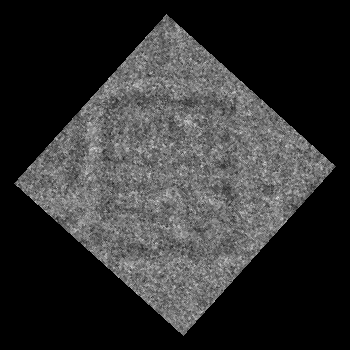 | 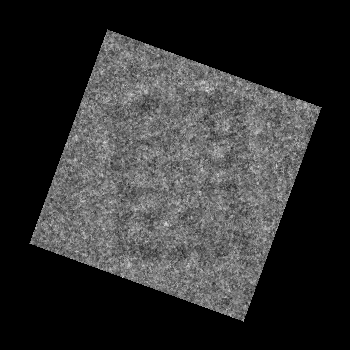 |

**Figure S6** Fully automated regular 2d side-view particles alignment results-based intensity-image registration and perfect generated particle masks using KLH dataset [22]

| 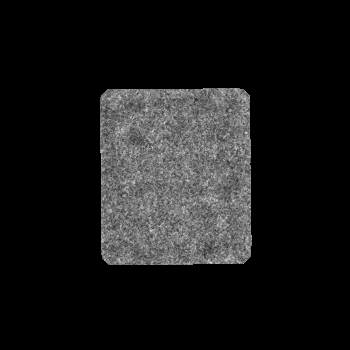 | 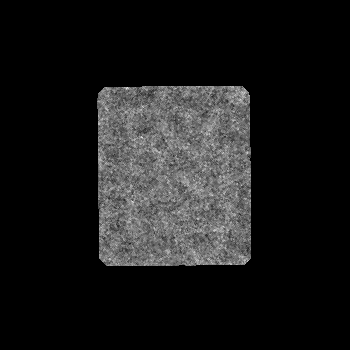 | 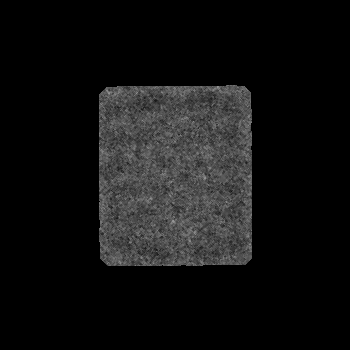 | 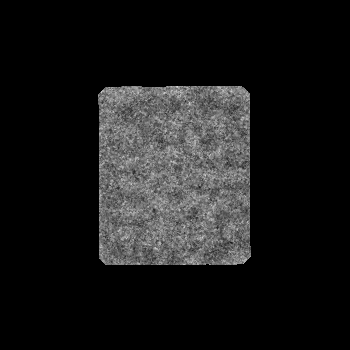 |
| --- | --- | --- | --- |
| (a) | | | |
| 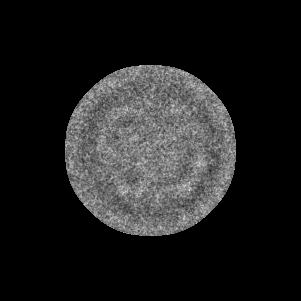 | 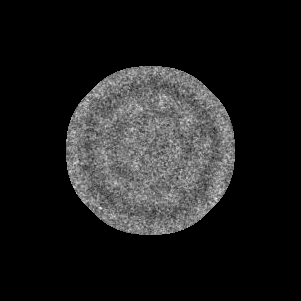 | 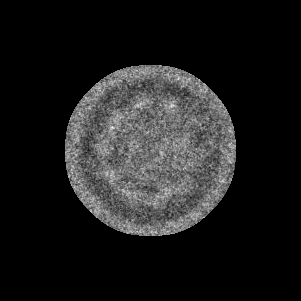 | 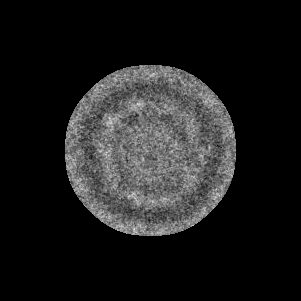 |
| (b) | | | |
| 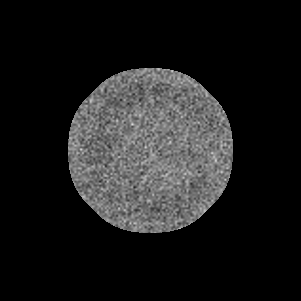 | 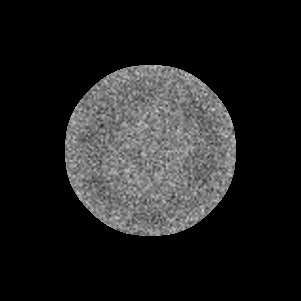 | 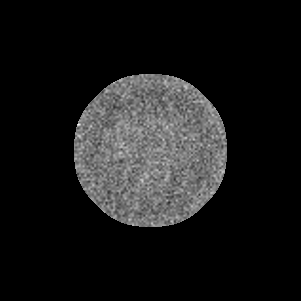 | 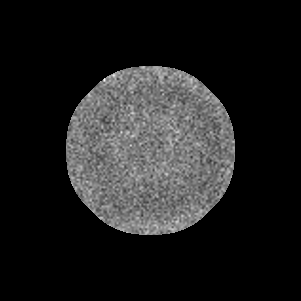 |
|  | | | |

**Figure S7** Fully automated localized 2d top and side-views particle alignment results using the original particles from KLH [22] and Apoferritin dataset [21].

| 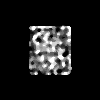 | 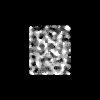 | 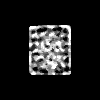 | 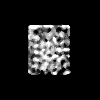 |
| --- | --- | --- | --- |
| (a) | | | |
| 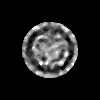 | 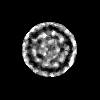 | 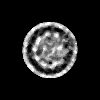 | 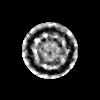 |
| (b) | | | |
| 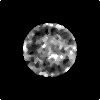 | 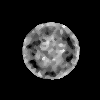 | 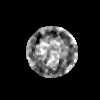 | 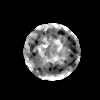 |
|  | | | |

**Figure S8** Fully automated localized 2d top and side-views particle alignment results using the pre-processed particles from KLH [22] and Apoferritin dataset [21].
